# Prospects for observing and localizing gravitational-wave transients with Advanced LIGO, Advanced Virgo and KAGRA

**DOI:** 10.1007/s41114-018-0012-9

**Published:** 2018-04-26

**Authors:** B. P. Abbott, R. Abbott, T. D. Abbott, M. R. Abernathy, F. Acernese, K. Ackley, C. Adams, T. Adams, P. Addesso, R. X. Adhikari, V. B. Adya, C. Affeldt, M. Agathos, K. Agatsuma, N. Aggarwal, O. D. Aguiar, L. Aiello, A. Ain, P. Ajith, T. Akutsu, B. Allen, A. Allocca, P. A. Altin, A. Ananyeva, S. B. Anderson, W. G. Anderson, M. Ando, S. Appert, K. Arai, A. Araya, M. C. Araya, J. S. Areeda, N. Arnaud, K. G. Arun, H. Asada, S. Ascenzi, G. Ashton, Y. Aso, M. Ast, S. M. Aston, P. Astone, S. Atsuta, P. Aufmuth, C. Aulbert, A. Avila-Alvarez, K. Awai, S. Babak, P. Bacon, M. K. M. Bader, L. Baiotti, P. T. Baker, F. Baldaccini, G. Ballardin, S. W. Ballmer, J. C. Barayoga, S. E. Barclay, B. C. Barish, D. Barker, F. Barone, B. Barr, L. Barsotti, M. Barsuglia, D. Barta, J. Bartlett, M. A. Barton, I. Bartos, R. Bassiri, A. Basti, J. C. Batch, C. Baune, V. Bavigadda, M. Bazzan, B. Bécsy, C. Beer, M. Bejger, I. Belahcene, M. Belgin, A. S. Bell, B. K. Berger, G. Bergmann, C. P. L. Berry, D. Bersanetti, A. Bertolini, J. Betzwieser, S. Bhagwat, R. Bhandare, I. A. Bilenko, G. Billingsley, C. R. Billman, J. Birch, R. Birney, O. Birnholtz, S. Biscans, A. Bisht, M. Bitossi, C. Biwer, M. A. Bizouard, J. K. Blackburn, J. Blackman, C. D. Blair, D. G. Blair, R. M. Blair, S. Bloemen, O. Bock, M. Boer, G. Bogaert, A. Bohe, F. Bondu, R. Bonnand, B. A. Boom, R. Bork, V. Boschi, S. Bose, Y. Bouffanais, A. Bozzi, C. Bradaschia, P. R. Brady, V. B. Braginsky, M. Branchesi, J. E. Brau, T. Briant, A. Brillet, M. Brinkmann, V. Brisson, P. Brockill, J. E. Broida, A. F. Brooks, D. A. Brown, D. D. Brown, N. M. Brown, S. Brunett, C. C. Buchanan, A. Buikema, T. Bulik, H. J. Bulten, A. Buonanno, D. Buskulic, C. Buy, R. L. Byer, M. Cabero, L. Cadonati, G. Cagnoli, C. Cahillane, J. Calderón Bustillo, T. A. Callister, E. Calloni, J. B. Camp, K. C. Cannon, H. Cao, J. Cao, C. D. Capano, E. Capocasa, F. Carbognani, S. Caride, J. Casanueva Diaz, C. Casentini, S. Caudill, M. Cavaglià, F. Cavalier, R. Cavalieri, G. Cella, C. B. Cepeda, L. Cerboni Baiardi, G. Cerretani, E. Cesarini, S. J. Chamberlin, M. Chan, S. Chao, P. Charlton, E. Chassande-Mottin, B. D. Cheeseboro, H. Y. Chen, Y. Chen, H.-P. Cheng, A. Chincarini, A. Chiummo, T. Chmiel, H. S. Cho, M. Cho, J. H. Chow, N. Christensen, Q. Chu, A. J. K. Chua, S. Chua, S. Chung, G. Ciani, F. Clara, J. A. Clark, F. Cleva, C. Cocchieri, E. Coccia, P.-F. Cohadon, A. Colla, C. G. Collette, L. Cominsky, M. Constancio, L. Conti, S. J. Cooper, T. R. Corbitt, N. Cornish, A. Corsi, S. Cortese, C. A. Costa, M. W. Coughlin, S. B. Coughlin, J.-P. Coulon, S. T. Countryman, P. Couvares, P. B. Covas, E. E. Cowan, D. M. Coward, M. J. Cowart, D. C. Coyne, R. Coyne, J. D. E. Creighton, T. D. Creighton, J. Cripe, S. G. Crowder, T. J. Cullen, A. Cumming, L. Cunningham, E. Cuoco, T. Dal Canton, S. L. Danilishin, S. D’Antonio, K. Danzmann, A. Dasgupta, C. F. Da Silva Costa, V. Dattilo, I. Dave, M. Davier, G. S. Davies, D. Davis, E. J. Daw, B. Day, R. Day, S. De, D. DeBra, G. Debreczeni, J. Degallaix, M. De Laurentis, S. Deléglise, W. Del Pozzo, T. Denker, T. Dent, V. Dergachev, R. De Rosa, R. T. DeRosa, R. DeSalvo, R. C. Devine, S. Dhurandhar, M. C. Díaz, L. Di Fiore, M. Di Giovanni, T. Di Girolamo, A. Di Lieto, S. Di Pace, I. Di Palma, A. Di Virgilio, Z. Doctor, K. Doi, V. Dolique, F. Donovan, K. L. Dooley, S. Doravari, I. Dorrington, R. Douglas, M. Dovale Álvarez, T. P. Downes, M. Drago, R. W. P. Drever, J. C. Driggers, Z. Du, M. Ducrot, S. E. Dwyer, K. Eda, T. B. Edo, M. C. Edwards, A. Effler, H.-B. Eggenstein, P. Ehrens, J. Eichholz, S. S. Eikenberry, R. A. Eisenstein, R. C. Essick, Z. Etienne, T. Etzel, M. Evans, T. M. Evans, R. Everett, M. Factourovich, V. Fafone, H. Fair, S. Fairhurst, X. Fan, S. Farinon, B. Farr, W. M. Farr, E. J. Fauchon-Jones, M. Favata, M. Fays, H. Fehrmann, M. M. Fejer, A. Fernández Galiana, I. Ferrante, E. C. Ferreira, F. Ferrini, F. Fidecaro, I. Fiori, D. Fiorucci, R. P. Fisher, R. Flaminio, M. Fletcher, H. Fong, S. S. Forsyth, J.-D. Fournier, S. Frasca, F. Frasconi, Z. Frei, A. Freise, R. Frey, V. Frey, E. M. Fries, P. Fritschel, V. V. Frolov, Y. Fujii, M.-K. Fujimoto, P. Fulda, M. Fyffe, H. Gabbard, B. U. Gadre, S. M. Gaebel, J. R. Gair, L. Gammaitoni, S. G. Gaonkar, F. Garufi, G. Gaur, V. Gayathri, N. Gehrels, G. Gemme, E. Genin, A. Gennai, J. George, L. Gergely, V. Germain, S. Ghonge, Abhirup Ghosh, Archisman Ghosh, S. Ghosh, J. A. Giaime, K. D. Giardina, A. Giazotto, K. Gill, A. Glaefke, E. Goetz, R. Goetz, L. Gondan, G. González, J. M. Gonzalez Castro, A. Gopakumar, M. L. Gorodetsky, S. E. Gossan, M. Gosselin, R. Gouaty, A. Grado, C. Graef, M. Granata, A. Grant, S. Gras, C. Gray, G. Greco, A. C. Green, P. Groot, H. Grote, S. Grunewald, G. M. Guidi, X. Guo, A. Gupta, M. K. Gupta, K. E. Gushwa, E. K. Gustafson, R. Gustafson, J. J. Hacker, A. Hagiwara, B. R. Hall, E. D. Hall, G. Hammond, M. Haney, M. M. Hanke, J. Hanks, C. Hanna, M. D. Hannam, J. Hanson, T. Hardwick, J. Harms, G. M. Harry, I. W. Harry, M. J. Hart, M. T. Hartman, C.-J. Haster, K. Haughian, K. Hayama, J. Healy, A. Heidmann, M. C. Heintze, H. Heitmann, P. Hello, G. Hemming, M. Hendry, I. S. Heng, J. Hennig, J. Henry, A. W. Heptonstall, M. Heurs, S. Hild, E. Hirose, D. Hoak, D. Hofman, K. Holt, D. E. Holz, P. Hopkins, J. Hough, E. A. Houston, E. J. Howell, Y. M. Hu, E. A. Huerta, D. Huet, B. Hughey, S. Husa, S. H. Huttner, T. Huynh-Dinh, N. Indik, D. R. Ingram, R. Inta, K. Ioka, H. N. Isa, J.-M. Isac, M. Isi, T. Isogai, Y. Itoh, B. R. Iyer, K. Izumi, T. Jacqmin, K. Jani, P. Jaranowski, S. Jawahar, F. Jiménez-Forteza, W. W. Johnson, D. I. Jones, R. Jones, R. J. G. Jonker, L. Ju, J. Junker, T. Kagawa, T. Kajita, M. Kakizaki, C. V. Kalaghatgi, V. Kalogera, M. Kamiizumi, N. Kanda, S. Kandhasamy, S. Kanemura, M. Kaneyama, G. Kang, J. B. Kanner, S. Karki, K. S. Karvinen, M. Kasprzack, Y. Kataoka, E. Katsavounidis, W. Katzman, S. Kaufer, T. Kaur, K. Kawabe, N. Kawai, S. Kawamura, F. Kéfélian, D. Keitel, D. B. Kelley, R. Kennedy, J. S. Key, F. Y. Khalili, I. Khan, S. Khan, Z. Khan, E. A. Khazanov, N. Kijbunchoo, C. Kim, H. Kim, J. C. Kim, J. Kim, W. Kim, Y.-M. Kim, S. J. Kimbrell, N. Kimura, E. J. King, P. J. King, R. Kirchhoff, J. S. Kissel, B. Klein, L. Kleybolte, S. Klimenko, P. Koch, S. M. Koehlenbeck, Y. Kojima, K. Kokeyama, S. Koley, K. Komori, V. Kondrashov, A. Kontos, M. Korobko, W. Z. Korth, K. Kotake, I. Kowalska, D. B. Kozak, C. Krämer, V. Kringel, B. Krishnan, A. Królak, G. Kuehn, P. Kumar, Rahul Kumar, Rakesh Kumar, L. Kuo, K. Kuroda, A. Kutynia, Y. Kuwahara, B. D. Lackey, M. Landry, R. N. Lang, J. Lange, B. Lantz, R. K. Lanza, A. Lartaux-Vollard, P. D. Lasky, M. Laxen, A. Lazzarini, C. Lazzaro, P. Leaci, S. Leavey, E. O. Lebigot, C. H. Lee, H. K. Lee, H. M. Lee, H. W. Lee, K. Lee, J. Lehmann, A. Lenon, M. Leonardi, J. R. Leong, N. Leroy, N. Letendre, Y. Levin, T. G. F. Li, A. Libson, T. B. Littenberg, J. Liu, N. A. Lockerbie, A. L. Lombardi, L. T. London, J. E. Lord, M. Lorenzini, V. Loriette, M. Lormand, G. Losurdo, J. D. Lough, C. O. Lousto, G. Lovelace, H. Lück, A. P. Lundgren, R. Lynch, Y. Ma, S. Macfoy, B. Machenschalk, M. MacInnis, D. M. Macleod, F. Magaña-Sandoval, E. Majorana, I. Maksimovic, V. Malvezzi, N. Man, V. Mandic, V. Mangano, S. Mano, G. L. Mansell, M. Manske, M. Mantovani, F. Marchesoni, M. Marchio, F. Marion, S. Márka, Z. Márka, A. S. Markosyan, E. Maros, F. Martelli, L. Martellini, I. W. Martin, D. V. Martynov, K. Mason, A. Masserot, T. J. Massinger, M. Masso-Reid, S. Mastrogiovanni, F. Matichard, L. Matone, N. Matsumoto, F. Matsushima, N. Mavalvala, N. Mazumder, R. McCarthy, D. E. McClelland, S. McCormick, C. McGrath, S. C. McGuire, G. McIntyre, J. McIver, D. J. McManus, T. McRae, S. T. McWilliams, D. Meacher, G. D. Meadors, J. Meidam, A. Melatos, G. Mendell, D. Mendoza-Gandara, R. A. Mercer, E. L. Merilh, M. Merzougui, S. Meshkov, C. Messenger, C. Messick, R. Metzdorff, P. M. Meyers, F. Mezzani, H. Miao, C. Michel, Y. Michimura, H. Middleton, E. E. Mikhailov, L. Milano, A. L. Miller, A. Miller, B. B. Miller, J. Miller, M. Millhouse, Y. Minenkov, J. Ming, S. Mirshekari, C. Mishra, V. P. Mitrofanov, G. Mitselmakher, R. Mittleman, O. Miyakawa, A. Miyamoto, T. Miyamoto, S. Miyoki, A. Moggi, M. Mohan, S. R. P. Mohapatra, M. Montani, B. C. Moore, C. J. Moore, D. Moraru, G. Moreno, W. Morii, S. Morisaki, Y. Moriwaki, S. R. Morriss, B. Mours, C. M. Mow-Lowry, G. Mueller, A. W. Muir, Arunava Mukherjee, D. Mukherjee, S. Mukherjee, N. Mukund, A. Mullavey, J. Munch, E. A. M. Muniz, P. G. Murray, A. Mytidis, S. Nagano, K. Nakamura, T. Nakamura, H. Nakano, Masaya Nakano, Masayuki Nakano, K. Nakao, K. Napier, I. Nardecchia, T. Narikawa, L. Naticchioni, G. Nelemans, T. J. N. Nelson, M. Neri, M. Nery, A. Neunzert, J. M. Newport, G. Newton, T. T. Nguyen, W.-T. Ni, A. B. Nielsen, S. Nissanke, A. Nitz, A. Noack, F. Nocera, D. Nolting, M. E. N. Normandin, L. K. Nuttall, J. Oberling, E. Ochsner, E. Oelker, G. H. Ogin, J. J. Oh, S. H. Oh, M. Ohashi, N. Ohishi, M. Ohkawa, F. Ohme, K. Okutomi, M. Oliver, K. Ono, Y. Ono, K. Oohara, P. Oppermann, Richard J. Oram, B. O’Reilly, R. O’Shaughnessy, D. J. Ottaway, H. Overmier, B. J. Owen, A. E. Pace, J. Page, A. Pai, S. A. Pai, J. R. Palamos, O. Palashov, C. Palomba, A. Pal-Singh, H. Pan, C. Pankow, F. Pannarale, B. C. Pant, F. Paoletti, A. Paoli, M. A. Papa, H. R. Paris, W. Parker, D. Pascucci, A. Pasqualetti, R. Passaquieti, D. Passuello, B. Patricelli, B. L. Pearlstone, M. Pedraza, R. Pedurand, L. Pekowsky, A. Pele, F. E. Peña Arellano, S. Penn, C. J. Perez, A. Perreca, L. M. Perri, H. P. Pfeiffer, M. Phelps, O. J. Piccinni, M. Pichot, F. Piergiovanni, V. Pierro, G. Pillant, L. Pinard, I. M. Pinto, M. Pitkin, M. Poe, R. Poggiani, P. Popolizio, A. Post, J. Powell, J. Prasad, J. W. W. Pratt, V. Predoi, T. Prestegard, M. Prijatelj, M. Principe, S. Privitera, G. A. Prodi, L. G. Prokhorov, O. Puncken, M. Punturo, P. Puppo, M. Pürrer, H. Qi, J. Qin, S. Qiu, V. Quetschke, E. A. Quintero, R. Quitzow-James, F. J. Raab, D. S. Rabeling, H. Radkins, P. Raffai, S. Raja, C. Rajan, M. Rakhmanov, P. Rapagnani, V. Raymond, M. Razzano, V. Re, J. Read, T. Regimbau, L. Rei, S. Reid, D. H. Reitze, H. Rew, S. D. Reyes, E. Rhoades, F. Ricci, K. Riles, M. Rizzo, N. A. Robertson, R. Robie, F. Robinet, A. Rocchi, L. Rolland, J. G. Rollins, V. J. Roma, R. Romano, J. H. Romie, D. Rosińska, S. Rowan, A. Rüdiger, P. Ruggi, K. Ryan, S. Sachdev, T. Sadecki, L. Sadeghian, N. Sago, M. Saijo, Y. Saito, K. Sakai, M. Sakellariadou, L. Salconi, M. Saleem, F. Salemi, A. Samajdar, L. Sammut, L. M. Sampson, E. J. Sanchez, V. Sandberg, J. R. Sanders, Y. Sasaki, B. Sassolas, B. S. Sathyaprakash, S. Sato, T. Sato, P. R. Saulson, O. Sauter, R. L. Savage, A. Sawadsky, P. Schale, J. Scheuer, E. Schmidt, J. Schmidt, P. Schmidt, R. Schnabel, R. M. S. Schofield, A. Schönbeck, E. Schreiber, D. Schuette, B. F. Schutz, S. G. Schwalbe, J. Scott, S. M. Scott, T. Sekiguchi, Y. Sekiguchi, D. Sellers, A. S. Sengupta, D. Sentenac, V. Sequino, A. Sergeev, Y. Setyawati, D. A. Shaddock, T. J. Shaffer, M. S. Shahriar, B. Shapiro, P. Shawhan, A. Sheperd, M. Shibata, Y. Shikano, T. Shimoda, A. Shoda, D. H. Shoemaker, D. M. Shoemaker, K. Siellez, X. Siemens, M. Sieniawska, D. Sigg, A. D. Silva, A. Singer, L. P. Singer, A. Singh, R. Singh, A. Singhal, A. M. Sintes, B. J. J. Slagmolen, B. Smith, J. R. Smith, R. J. E. Smith, K. Somiya, E. J. Son, B. Sorazu, F. Sorrentino, T. Souradeep, A. P. Spencer, A. K. Srivastava, A. Staley, M. Steinke, J. Steinlechner, S. Steinlechner, D. Steinmeyer, B. C. Stephens, S. P. Stevenson, R. Stone, K. A. Strain, N. Straniero, G. Stratta, S. E. Strigin, R. Sturani, A. L. Stuver, Y. Sugimoto, T. Z. Summerscales, L. Sun, S. Sunil, P. J. Sutton, T. Suzuki, B. L. Swinkels, M. J. Szczepańczyk, M. Tacca, H. Tagoshi, S. Takada, H. Takahashi, R. Takahashi, A. Takamori, D. Talukder, H. Tanaka, K. Tanaka, T. Tanaka, D. B. Tanner, M. Tápai, A. Taracchini, D. Tatsumi, R. Taylor, S. Telada, T. Theeg, E. G. Thomas, M. Thomas, P. Thomas, K. A. Thorne, E. Thrane, T. Tippens, S. Tiwari, V. Tiwari, K. V. Tokmakov, K. Toland, T. Tomaru, C. Tomlinson, M. Tonelli, Z. Tornasi, C. I. Torrie, D. Töyrä, F. Travasso, G. Traylor, D. Trifirò, J. Trinastic, M. C. Tringali, L. Trozzo, M. Tse, R. Tso, K. Tsubono, T. Tsuzuki, M. Turconi, D. Tuyenbayev, T. Uchiyama, T. Uehara, S. Ueki, K. Ueno, D. Ugolini, C. S. Unnikrishnan, A. L. Urban, T. Ushiba, S. A. Usman, H. Vahlbruch, G. Vajente, G. Valdes, N. van Bakel, M. van Beuzekom, J. F. J. van den Brand, C. Van Den Broeck, D. C. Vander-Hyde, L. van der Schaaf, J. V. van Heijningen, M. H. P. M. van Putten, A. A. van Veggel, M. Vardaro, V. Varma, S. Vass, M. Vasúth, A. Vecchio, G. Vedovato, J. Veitch, P. J. Veitch, K. Venkateswara, G. Venugopalan, D. Verkindt, F. Vetrano, A. Viceré, A. D. Viets, S. Vinciguerra, D. J. Vine, J.-Y. Vinet, S. Vitale, T. Vo, H. Vocca, C. Vorvick, D. V. Voss, W. D. Vousden, S. P. Vyatchanin, A. R. Wade, L. E. Wade, M. Wade, T. Wakamatsu, M. Walker, L. Wallace, S. Walsh, G. Wang, H. Wang, M. Wang, Y. Wang, R. L. Ward, J. Warner, M. Was, J. Watchi, B. Weaver, L.-W. Wei, M. Weinert, A. J. Weinstein, R. Weiss, L. Wen, P. Weßels, T. Westphal, K. Wette, J. T. Whelan, B. F. Whiting, C. Whittle, D. Williams, R. D. Williams, A. R. Williamson, J. L. Willis, B. Willke, M. H. Wimmer, W. Winkler, C. C. Wipf, H. Wittel, G. Woan, J. Woehler, J. Worden, J. L. Wright, D. S. Wu, G. Wu, W. Yam, H. Yamamoto, K. Yamamoto, T. Yamamoto, C. C. Yancey, K. Yano, M. J. Yap, J. Yokoyama, T. Yokozawa, T. H. Yoon, Hang Yu, Haocun Yu, H. Yuzurihara, M. Yvert, A. Zadrożny, L. Zangrando, M. Zanolin, S. Zeidler, J.-P. Zendri, M. Zevin, L. Zhang, M. Zhang, T. Zhang, Y. Zhang, C. Zhao, M. Zhou, Z. Zhou, S. J. Zhu, X. J. Zhu, M. E. Zucker, J. Zweizig, KAGRA Collaboration, LIGO Scientific Collaboration and Virgo Collaboration

**Affiliations:** 1 LIGO, California Institute of Technology, Pasadena, CA 91125 USA; 2 Louisiana State University, Baton Rouge, LA 70803 USA; 3 American University, Washington, DC 20016 USA; 4 Università di Salerno, Fisciano, I-84084 Salerno Italy; 5 INFN, Sezione di Napoli, Complesso Universitario di Monte S.Angelo, I-80126 Napoli, Italy; 6 University of Florida, Gainesville, FL 32611 USA; 7 LIGO Livingston Observatory, Livingston, LA 70754 USA; 8 Laboratoire d’Annecy-le-Vieux de Physique des Particules (LAPP), Université Savoie Mont Blanc, CNRS/IN2P3, F-74941 Annecy-le-Vieux, France; 9 University of Sannio at Benevento, I-82100 Benevento, Italy; 10 INFN, Sezione di Napoli, I-80100 Napoli, Italy; 11 Albert-Einstein-Institut, Max-Planck-Institut für Gravitationsphysik, D-30167 Hannover, Germany; 12 Nikhef, Science Park, 1098 XG Amsterdam, The Netherlands; 13 LIGO, Massachusetts Institute of Technology, Cambridge, MA 02139 USA; 14 Instituto Nacional de Pesquisas Espaciais, 12227-010 São José dos Campos, São Paulo Brazil; 15 INFN, Gran Sasso Science Institute, I-67100 L’Aquila, Italy; 16 INFN, Sezione di Roma Tor Vergata, I-00133 Roma, Italy; 17 Inter-University Centre for Astronomy and Astrophysics, Pune, 411007 India; 18 International Centre for Theoretical Sciences, Tata Institute of Fundamental Research, Bengaluru, 560089 India; 19 National Astronomical Observatory of Japan, 2-21-1, Ohsawa, Mitaka-shi, Tokyo 181-8588 Japan; 20 University of Wisconsin-Milwaukee, Milwaukee, Wisconsin 53201 USA; 21 Leibniz Universität Hannover, D-30167 Hannover, Germany; 22 Università di Pisa, I-56127 Pisa, Italy; 23 INFN, Sezione di Pisa, I-56127 Pisa, Italy; 24 Australian National University, Canberra, Australian Capital Territory 0200 Australia; 25 The University of Tokyo, Department of Physics, 7-3-1, Hongo, Bunkyo-ku, Tokyo 113-0033 Japan; 26 The University of Tokyo, Research Center for the Early Universe, 7-3-1, Hongo, Bunkyo-ku, Tokyo 113-0033 Japan; 27 The University of Tokyo, Earthquake Research Institute, 1-1-1, Yayoi, Bunkyo-ku, Tokyo 113-0032 Japan; 28 California State University Fullerton, Fullerton, CA 92831 USA; 29 LAL, Univ. Paris-Sud, CNRS/IN2P3, Université Paris-Saclay, F-91898 Orsay, France; 30 Chennai Mathematical Institute, Chennai, 603103 India; 31 Hirosaki University, Department of Advanced Physics, 3, Bunkyo-cho, Hirosaki-shi, Aomori 036-8561 Japan; 32 Università di Roma Tor Vergata, I-00133 Roma, Italy; 33 Universität Hamburg, D-22761 Hamburg, Germany; 34 INFN, Sezione di Roma, I-00185 Roma, Italy; 35 Tokyo Institute of Technology, Graduate School of Science and Technology, 2-12-1, Ookayama, Meguro-ku, Tokyo 152-8551 Japan; 36 The University of Tokyo, Institute for Cosmic Ray Research, Higashi-Mozumi 238, Kamioka-cho, Hida-shi, Gifu 506-1205 Japan; 37 Albert-Einstein-Institut, Max-Planck-Institut für Gravitationsphysik, D-14476 Potsdam-Golm, Germany; 38 APC, AstroParticule et Cosmologie, Université Paris Diderot, CNRS/IN2P3, CEA/Irfu, Observatoire de Paris, Sorbonne Paris Cité, F-75205 Paris Cedex 13 France; 39 Osaka University, Graduate School of Science, Physics, 1-1, Machikaneyama-cho, Toyonaka-shi, Osaka 560-0043 Japan; 40 West Virginia University, Morgantown, WV 26506 USA; 41 Center for Gravitational Waves and Cosmology, West Virginia University, Morgantown, WV 26505 USA; 42 Università di Perugia, I-06123 Perugia, Italy; 43 INFN, Sezione di Perugia, I-06123 Perugia, Italy; 44 European Gravitational Observatory (EGO), I-56021 Cascina, Pisa Italy; 45 Syracuse University, Syracuse, NY 13244 USA; 46 SUPA, University of Glasgow, Glasgow, G12 8QQ United Kingdom; 47 LIGO Hanford Observatory, Richland, WA 99352 USA; 48 Wigner RCP, RMKI, Konkoly Thege Miklós út 29-33, H-1121 Budapest, Hungary; 49 Columbia University, New York, NY 10027 USA; 50 Stanford University, Stanford, CA 94305 USA; 51 Università di Padova, Dipartimento di Fisica e Astronomia, I-35131 Padova, Italy; 52 INFN, Sezione di Padova, I-35131 Padova, Italy; 53 MTA Eötvös University, “Lendulet” Astrophysics Research Group, Budapest, 1117 Hungary; 54 Nicolaus Copernicus Astronomical Center, Polish Academy of Sciences, 00-716 Warsaw, Poland; 55 Center for Relativistic Astrophysics and School of Physics, Georgia Institute of Technology, Atlanta, GA 30332 USA; 56 University of Birmingham, Birmingham, B15 2TT United Kingdom; 57 Università degli Studi di Genova, I-16146 Genova, Italy; 58 INFN, Sezione di Genova, I-16146 Genova, Italy; 59 RRCAT, Indore, MP 452013 India; 60 Faculty of Physics, Lomonosov Moscow State University, Moscow, 119991 Russia; 61 SUPA, University of the West of Scotland, Paisley, PA1 2BE United Kingdom; 62 Caltech CaRT, Pasadena, CA 91125 USA; 63 University of Western Australia, Crawley, Western Australia 6009 Australia; 64 Department of Astrophysics/IMAPP, Radboud University Nijmegen, P.O. Box 9010, 6500 GL Nijmegen, The Netherlands; 65 Artemis, Université Côte d’Azur, CNRS, Observatoire Côte d’Azur, CS 34229, F-06304 Nice Cedex 4, France; 66 Institut de Physique de Rennes, CNRS, Université de Rennes 1, F-35042 Rennes, France; 67 Washington State University, Pullman, WA 99164 USA; 68 Università degli Studi di Urbino ’Carlo Bo’, I-61029 Urbino, Italy; 69 INFN, Sezione di Firenze, I-50019 Sesto Fiorentino, Firenze Italy; 70 University of Oregon, Eugene, OR 97403 USA; 71 Laboratoire Kastler Brossel, UPMC-Sorbonne Universités, CNRS, ENS-PSL Research University, Collège de France, F-75005 Paris, France; 72 Carleton College, Northfield, MN 55057 USA; 73 Astronomical Observatory Warsaw University, 00-478 Warsaw, Poland; 74 VU University Amsterdam, 1081 HV Amsterdam, The Netherlands; 75 University of Maryland, College Park, MD 20742 USA; 76 Laboratoire des Matériaux Avancés (LMA), CNRS/IN2P3, F-69622 Villeurbanne, France; 77 Université Claude Bernard Lyon 1, F-69622 Villeurbanne, France; 78 Università di Napoli ’Federico II’, Complesso Universitario di Monte S.Angelo, I-80126 Napoli, Italy; 79 NASA/Goddard Space Flight Center, Greenbelt, MD 20771 USA; 80 University of Adelaide, Adelaide, South Australia 5005 Australia; 81 Tsinghua University, Beijing, 100084 China; 82 Texas Tech University, Lubbock, TX 79409 USA; 83 The University of Mississippi, University, MS 38677 USA; 84 The Pennsylvania State University, University Park, PA 16802 USA; 85 National Tsing Hua University, Hsinchu City, 30013 Taiwan Republic of China; 86 Charles Sturt University, Wagga Wagga, New South Wales 2678 Australia; 87 University of Chicago, Chicago, IL 60637 USA; 88 Kenyon College, Gambier, OH 43022 USA; 89 Korea Institute of Science and Technology Information, Daejeon, 34141 Korea; 90 University of Cambridge, Cambridge, CB2 1TN United Kingdom; 91 Università di Roma ’La Sapienza’, I-00185 Roma, Italy; 92 Université Libre de Bruxelles, Brussels, 1050 Belgium; 93 Sonoma State University, Rohnert Park, CA 94928 USA; 94 Montana State University, Bozeman, MT 59717 USA; 95 Center for Interdisciplinary Exploration & Research in Astrophysics (CIERA), Northwestern University, Evanston, IL 60208 USA; 96 Universitat de les Illes Balears, IAC3—IEEC, E-07122 Palma de Mallorca, Spain; 97 The University of Texas Rio Grande Valley, Brownsville, TX 78520 USA; 98 Bellevue College, Bellevue, WA 98007 USA; 99 Institute for Plasma Research, Bhat, Gandhinagar 382428 India; 100 The University of Sheffield, Sheffield, S10 2TN United Kingdom; 101 California State University, Los Angeles, 5154 State University Dr, Los Angeles, CA 90032 USA; 102 Università di Trento, Dipartimento di Fisica, I-38123 Povo, Trento Italy; 103 INFN, Trento Institute for Fundamental Physics and Applications, I-38123 Povo, Trento Italy; 104 University of Toyama, 3190 Gofuku, Toyama-shi, Toyama 930-8555 Japan; 105 Cardiff University, Cardiff, CF24 3AA United Kingdom; 106 Montclair State University, Montclair, NJ 07043 USA; 107 Canadian Institute for Theoretical Astrophysics, University of Toronto, Toronto, Ontario M5S 3H8 Canada; 108 School of Mathematics, University of Edinburgh, Edinburgh, EH9 3FD United Kingdom; 109 University and Institute of Advanced Research, Gandhinagar, Gujarat 382007 India; 110 IISER-TVM, CET Campus, Trivandrum Kerala, 695016 India; 111 University of Szeged, Dóm tér 9, Szeged, 6720 Hungary; 112 Embry-Riddle Aeronautical University, Prescott, AZ 86301 USA; 113 Tata Institute of Fundamental Research, Mumbai, 400005 India; 114 INAF, Osservatorio Astronomico di Capodimonte, I-80131 Napoli, Italy; 115 University of Michigan, Ann Arbor, MI 48109 USA; 116 High Energy Accelerator Research Organization, 1-1, Oho, Tsukuba-shi, Ibaraki 305-0801 Japan; 117 Rochester Institute of Technology, Rochester, NY 14623 USA; 118 NCSA, University of Illinois at Urbana-Champaign, Urbana, IL 61801 USA; 119 Center for Gravitational Physics, Yukawa Institute for Theoretical Physics, Kyoto University, Kyoto, 606-8502 Japan; 120 University of Białystok, 15-424 Białystok, Poland; 121 SUPA, University of Strathclyde, Glasgow, G1 1XQ United Kingdom; 122 University of Southampton, Southampton, SO17 1BJ United Kingdom; 123 Osaka City University, Department of Physics, 3-3-138, Sugimoto-cho, Sumiyosi-ku, Osaka-shi, Osaka 558-8585 Japan; 124 University of Washington Bothell, 18115 Campus Way NE, Bothell, WA 98011 USA; 125 Institute of Applied Physics, Nizhny Novgorod, 603950 Russia; 126 Korea Astronomy and Space Science Institute (KASI), 776, Daedeokdae-ro, Yuseong-gu, Daejeon 34055 Republic of Korea; 127 National Institute for Mathematical Sciences, Daejeon, 34047 Korea; 128 Inje University, 197 Inje-ro, Gimhae-si, 50834 Korea; 129 Myongji University, Yongin, 449-728 Korea; 130 Pusan National University, Busan, 609-735 Korea; 131 Seoul National University, Seoul, 151-742 Korea; 132 Hiroshima University, Department of Physical Science, 1-3-1, Kagamiyama, Higashihiroshima-shi, Hiroshima 739-8526 Japan; 133 Department of Applied Physics, Fukuoka University, Fukuoka, Jonan, Nanakuma 814-0180 Japan; 134 NCBJ, 05-400 Świerk-Otwock, Poland; 135 Institute of Mathematics, Polish Academy of Sciences, 00656 Warsaw, Poland; 136 The School of Physics & Astronomy, Monash University, Clayton, 3800 Victoria Australia; 137 Hanyang University, Seoul, 133-791 Korea; 138 The Chinese University of Hong Kong, Shatin, NT Hong Kong; 139 University of Alabama in Huntsville, Huntsville, AL 35899 USA; 140 ESPCI, CNRS, F-75005 Paris, France; 141 University of Minnesota, Minneapolis, MN 55455 USA; 142 The Institute of Statistical Mathematics, Department of Mathematical Analysis and Statistical Inference, 10-3 Midori-cho, Tachikawa, Tokyo 190-8562 Japan; 143 Università di Camerino, Dipartimento di Fisica, I-62032 Camerino, Italy; 144 Tohoku University, Sendai, Miyagi 982-0826 Japan; 145 Southern University and A&M College, Baton Rouge, LA 70813 USA; 146 The University of Melbourne, Parkville, Victoria 3010 Australia; 147 College of William and Mary, Williamsburg, VA 23187 USA; 148 Instituto de Física Teórica, University Estadual Paulista/ICTP South American Institute for Fundamental Research, São Paulo, SP 01140-070 Brazil; 149 The Kyoto University, Disaster Prevention Research Institute, Gokasho, Uji, Kyoto 611-0011 Japan; 150 National Institute of Information and Communications Technology, The Applied Electromagnetic Research Institute , 4-2-1, Nukuikita-machi, Koganei-shi, Tokyo 184-8795 Japan; 151 Kyoto University, Department of Physics, Astronomy, Oiwake-cho, KitaShirakawa, Sakyou-ku, Kyoto-shi, Kyoto 606-8502 Japan; 152 National Tsing Hua University, Department of Physics, No. 101, Section 2, Kuang-Fu Road, Hsinchu, Taiwan 30013 ROC; 153 University of Shanghai for Science and Technology, School of Optical-Electrical and Computer Engineering, 516, Jun Gong Rd, Shanghai, 200093 P. R. China; 154 Whitman College, 345 Boyer Avenue, Walla Walla, WA 99362 USA; 155 Niigata University, Faculty of Engineering, 8050, Ikarashi-2-no-cho, Nishi-ku, Niigata-shi, Niigata 950-2181 Japan; 156 Sokendai (The Graduate University for Advanced Studies), 2-21-1, Ohsawa, Mitaka-shi, Tokyo 181-8588 Japan; 157 Niigata University, Graduate School of Science and Technology, 8050, Ikarashi-2-no-cho, Nishi-ku, Niigata-shi, Niigata, 950-2181 Japan; 158 Université de Lyon, F-69361 Lyon, France; 159 Hobart and William Smith Colleges, Geneva, NY 14456 USA; 160 Janusz Gil Institute of Astronomy, University of Zielona Góra, 65-265 Zielona Góra, Poland; 161 Kyushu University, Faculty of Arts and Science, 744, Motooka, Nishi-ku, Fukuoka 819-0395 Japan; 162 Waseda University, Department of Physics, 3-4-1, Okubo, Shinjuku, Tokyo 169-8555 Japan; 163 Nagaoka University of Technology, Department of Information Science and Control Engineering, 1603-1 Kamitomioka, Nagaoka, Niigata 940-2188 Japan; 164 King’s College London, University of London, London, WC2R 2LS United Kingdom; 165 IISER-Kolkata, Mohanpur, West Bengal 741252 India; 166 Nagaoka University of Technology, Department of Information & Management Systems Engineering, 1603-1 Kamitomioka, Nagaoka, Niigata 940-2188 Japan; 167 Hosei University, The Graduate School of Science and Engineering, Kajino-cho 3-7-2, Koganei-shi, Tokyo 184-8584 Japan; 168 Toho University, Faculty of Science, 2-2-1 Miyama, Funabashi-shi, Chiba Japan; 169 Indian Institute of Technology, Gandhinagar Ahmedabad, Gujarat 382424 India; 170 Institute for Molecular Science, National Institutes of Natural Sciences, 38 Nishigo-Naka, Myodaiji, Okazaki 444-8585 Japan; 171 Institute for Quantum Studies, Chapman University, 1 University Dr, Orange, CA 92866 USA; 172 Andrews University, Berrien Springs, MI 49104 USA; 173 National Institutes of Natural Sciences, The Device Engineering and Applied Physics Research Division, 322-6 Oroshi-cho, Toki city, GIFU Prefecture 509-5292 Japan; 174 National Institute of Advanced Industrial Science and Technology, Metrology Institute of Japan, 1-1-1, Umezono, Tsukuba-shi, Ibaraki 305-8568 Japan; 175 Università di Siena, I-53100 Siena, Italy; 176 National Defense Academy of Japan, Department of Communications Engineering, Hashirimizu 1-10-20, Yokosuka-shi, Kanagawa-Pref 239-8686 Japan; 177 Trinity University, San Antonio, TX 78212 USA; 178 Physics and Astronomy, Sejong University, 209 Neungdong-ro, Gwangjin-gu, 143-747 Seoul South Korea; 179 University of Washington, Seattle, WA 98195 USA; 180 Abilene Christian University, Abilene, TX 79699 USA; 181 Department of Physics, Korea University, 145, Anam-ro, Seongbuk-gu, Seoul 02841 Korea

**Keywords:** Gravitational waves, Gravitational-wave detectors, Electromagnetic counterparts, Data analysis

## Abstract

We present possible observing scenarios for the Advanced LIGO, Advanced Virgo and KAGRA gravitational-wave detectors over the next decade, with the intention of providing information to the astronomy community to facilitate planning for multi-messenger astronomy with gravitational waves. We estimate the sensitivity of the network to transient gravitational-wave signals, and study the capability of the network to determine the sky location of the source. We report our findings for gravitational-wave transients, with particular focus on gravitational-wave signals from the inspiral of binary neutron star systems, which are the most promising targets for multi-messenger astronomy. The ability to localize the sources of the detected signals depends on the geographical distribution of the detectors and their relative sensitivity, and $90\%$ credible regions can be as large as thousands of square degrees when only two sensitive detectors are operational. Determining the sky position of a significant fraction of detected signals to areas of 5–$20~\mathrm {deg}^2$ requires at least three detectors of sensitivity within a factor of $\sim 2$ of each other and with a broad frequency bandwidth. When all detectors, including KAGRA and the third LIGO detector in India, reach design sensitivity, a significant fraction of gravitational-wave signals will be localized to a few square degrees by gravitational-wave observations alone.

## Introduction

Advanced LIGO (aLIGO; Harry 2010; Aasi et al. 2015a), Advanced Virgo (AdV; Acernese et al. 2009; Accadia et al. 2012; Acernese et al. 2015) and KAGRA (Somiya 2012; Aso et al. 2013) are kilometer-scale gravitational-wave (GW) detectors that are sensitive to GWs with frequencies of $\sim 20$–$2000~\mathrm {Hz}$.^1^ The era of GW astronomy began with the detection of GW150914 (Abbott et al. 2016j), a signal from the coalescence of a binary black hole (BBH); the first confirmed multi-messenger counterpart to a GW observation came with GW170817 (Abbott et al. 2017i), a signal from a binary neutron star (BNS) coalescence which was accompanied by detections across the electromagnetic spectrum (Abbott et al. 2017k). In this article, we describe the currently projected schedule, sensitivity, and sky-localization accuracy for the GW-detector network. We discuss the past and future planned sequence of observing runs (designated O1, O2, O3, etc.) and the prospects for multi-messenger astronomy.

The purpose of this article is to provide information to the astronomy community to assist in the formulation of plans for forthcoming GW observations. In particular, we intend this article to provide the information required for assessing the features of programs for joint observation of GW events using electromagnetic, neutrino, or other facilities (e.g., Abbott et al. 2016i; Adrian-Martinez et al. 2016; Albert et al. 2017c; Abbott et al. 2017k).

The full science of ground-based GW detectors is broad (Abbott et al. 2016j), and is not covered in this article. We concentrate solely on candidate GW transient signals. We place particular emphasis on the coalescence of BNS systems, which are the GW source for which electromagnetic follow-up is most promising (Metzger and Berger 2012; Patricelli et al. 2016; Paschalidis 2017; Rosswog et al. 2017; Metzger 2017). However, we also mention BBHs, as they are the most commonly detected source (Abbott et al. 2016c, 2017f). No electromagnetic emission is expected for vacuum BBH mergers (Centrella et al. 2010), but is possible if there is surrounding material (Schnittman 2013), for example, remnants of mass lost from the parent star (Perna et al. 2016; Janiuk et al. 2017) or if the binary was embedded in a circumbinary disc or a common envelope (Bartos et al. 2017; Woosley 2016; Stone et al. 2017). For more general introductory articles on GW generation, detection and astrophysics, we point readers to Blanchet (2014), Pitkin et al. (2011), Sathyaprakash and Schutz (2009).

Although our collaborations have amassed a great deal of experience with GW detectors and analysis, it is still difficult to make predictions for both improvements in search methods and for the rate of progress for detectors which are not yet fully installed or operational. *The scenarios of detector sensitivity evolution and observing times given here should not be considered as fixed or firm commitments.*

As the detectors’ construction and commissioning progress, we intend to release updated versions of this article. This is the third version of the article, written to coincide with the close of the second observing run (O2) of the advanced-detector era. Changes with respect to the previous version (Aasi et al. 2016) are given in Appendix A. Progress has been made in the commissioning of the detectors. We include projections for KAGRA for the first time; we also include results from the first observing run (O1) and currently available results from O2.

## Commissioning and observing phases

We divide the development of the GW observatories into three components:

- Construction includes the installation and testing of the detectors. This phase ends with *acceptance* of the detectors. Acceptance means that the interferometers can lock for periods of hours: light is resonant in the arms of the interferometer with *no guaranteed GW sensitivity.* Construction incorporates several short *engineering runs* with no astrophysical output as the detectors progress towards acceptance. The aLIGO construction project ended in March 2015. The construction of AdV was completed at the end of 2016. KAGRA will be operational in its full configuration by early 2019.
- Commissioning improves the detectors’ performance with the goal of reaching the design sensitivity. Engineering runs in the commissioning phase allow us to understand our detectors and analyses in an observational mode; these are not intended to produce astrophysical results, but that does not preclude the possibility of this happening.^2^ Rather than proceeding directly to design sensitivity before making astrophysical observations, commissioning is interleaved with *observing runs*.
- Observing runs begin when the detectors have reached (and can stably maintain) a significantly improved sensitivity compared with previous operation. Observing runs will produce astrophysical results, direct detections from some GW sources and upper limits on the rates or energetics of others. During the first two observing runs (O1 and O2), exchange of GW candidates with partners outside the LIGO Scientific Collaboration (LSC) and the Virgo Collaboration was governed by memoranda of understanding (MOUs; Abadie et al. 2012d; Aasi et al. 2013b). From the start of the third observing run (O3), all GW event candidates identified with high confidence will be released immediately to the full astronomical community. KAGRA will become a part of the global network with full data sharing, once sensitivities comparable with aLIGO and AdV are achieved.

The progress in sensitivity as a function of time will influence the duration of the observing runs that we plan at any stage. Commissioning is a complex process which involves both scheduled improvements to the detectors and tackling unexpected new problems. While our experience makes us cautiously optimistic regarding the schedule for the advanced detectors, we are targeting an order of magnitude improvement in sensitivity relative to the previous generation of detectors over a wider frequency band. Consequently, it is not possible to make concrete predictions for sensitivity or duty cycle as a function of time. We can, however, use our experience as a guide to plausible scenarios for the detector operational states that will allow us to reach the desired sensitivity. Unexpected problems could slow down the commissioning, but there is also the possibility that progress may happen faster than predicted here. The schedule of commissioning phases and observation runs will be driven by a cost–benefit analysis of the time required to make significant sensitivity improvements. More information on event rates could also change the schedule and duration of runs.

In Sect. 2.1, we present the commissioning plans for the aLIGO, AdV and KAGRA detectors. A summary of expected observing runs is in Sect. 2.2.

**Fig. 1:**
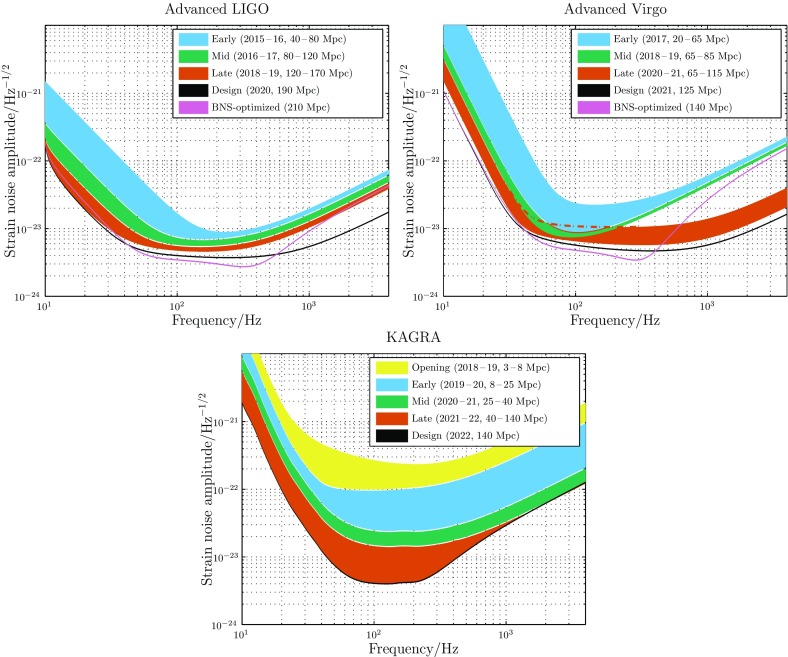
Regions of aLIGO (*top left*), AdV (*top right*) and KAGRA (*bottom*) target strain sensitivities as a function of frequency. The binary neutron star (BNS) range, the average distance to which these signals could be detected, is given in megaparsec. Current notions of the progression of sensitivity are given for early, mid and late commissioning phases, as well as the design sensitivity target and the BNS-optimized sensitivity. While both dates and sensitivity curves are subject to change, the overall progression represents our best current estimates

### Commissioning and observing roadmap

The anticipated strain sensitivity evolution for aLIGO, AdV and KAGRA is shown in Fig. 1. As a standard figure of merit for detector sensitivity, we use the range, the volume- and orientation-averaged distance at which a compact binary coalescence consisting of a particular mass gives a matched filter signal-to-noise ratio (SNR) of 8 in a single detector (Finn and Chernoff 1993). We define $V_z$ as the orientation-averaged spacetime volume surveyed per unit detector time; for a population with a constant comoving source-frame rate density, $V_z$ multiplied by the rate density gives the detection rate of those sources by the particular detector. We define the range *R* as the distance for which $(4\pi /3){R}^3 = {V_z}$. In Table 1 we present values of *R* for different detector networks and binary sources. For further insight into the range, and a discussion of additional quantities such as the median and average distances to sources, please see Chen et al. (2017). The BNS ranges, assuming two $1.4\,M_\odot$ neutron stars, for the various stages of the expected evolution are provided in Fig. 1, and the BNS and BBH ranges are quoted in Table 1.

**Table 1:** Plausible target detector sensitivities

	LIGO		Virgo		KAGRA	
	BNS	BBH	BNS	BBH	BNS	BBH
	range/$\mathrm {Mpc}$	range/$\mathrm {Mpc}$	range/$\mathrm {Mpc}$	range/$\mathrm {Mpc}$	range/$\mathrm {Mpc}$	range/$\mathrm {Mpc}$
Early	40–80	415–775	20–65	220–615	8–25	8–250
Mid	80–120	775–1110	65–85	615–790	25–40	250–405
Late	120–170	1110–1490	65–115	610–1030	40–140	405–1270
Design	190	1640	125	1130	140	1270

The different phases match those in Fig. 1. We quote the range, the average distance to which a signal could be detected, for a $1.4\,M_{\odot }$+$1.4\,M_{\odot }$ binary neutron star (BNS) system and a $30\,M_{\odot }$+$30\,M_{\odot }$ binary black hole (BBH) system

There are currently two operational aLIGO detectors (Aasi et al. 2015a). The original plan called for three identical 4-$\mathrm {km}$ interferometers, two at Hanford (H1 and H2) and one at Livingston (L1). In 2011, the LIGO Lab and IndIGO consortium in India proposed installing one of the aLIGO Hanford detectors (H2) at a new observatory in India (LIGO-India; Iyer et al. 2011). In early 2015, LIGO Laboratory placed the H2 interferometer in long-term storage for use in India. The Government of India granted in-principle approval to LIGO-India in February 2016.

The first observations with aLIGO have been made. O1 formally began 18 September 2015 and ended 12 January 2016; however, data from the surrounding engineering periods were of sufficient quality to be included in the analysis, and hence the first observations span 12 September 2015 to 19 January 2016. The run involved the H1 and L1 detectors; the detectors were not at full design sensitivity (Abbott et al. 2016f). We aimed for a BNS range of 40–80 $\mathrm {Mpc}$ for both instruments (see Fig. 1), and achieved a 60–80 $\mathrm {Mpc}$ range. Subsequent observing runs have increasing duration and sensitivity. O2 began 30 November 2016, transitioning from the preceding engineering run which began at the end of October, and ended 25 August 2017. The achieved sensitivity across the run was typically in the range 60–100 $\mathrm {Mpc}$ (Abbott et al. 2017f). Several improvements to the aLIGO detectors will be performed between O2 and O3, including further mitigation of technical noises, increase of laser power delivered to the interferometer, and installation of a squeezed vacuum source. Assuming that no unexpected obstacles are encountered, the aLIGO detectors are expected to achieve a 190 $\mathrm {Mpc}$ BNS range by 2020. After the first observing runs, it might be desirable to optimize the detector sensitivity for a specific class of astrophysical signals, such as BNSs. The BNS range may then become 210 $\mathrm {Mpc}$. The sensitivity for each of these stages is shown in Fig. 1.

The H2 detector will be installed in India once the LIGO-India Observatory is completed, and will be configured to be identical to the H1 and L1 detectors. We refer to the detector in this state as I1 (rather than H2). Operation at the same level as the H1 and L1 detectors is anticipated for no earlier than 2024.

The AdV interferometer (V1; Accadia et al. 2012) officially joined O2 on 1 August 2017. We aimed for an early step with sensitivity corresponding to a BNS range of 20–65 $\mathrm {Mpc}$; however, AdV used steel wires to suspend the test masses, instead of fused silica fibers. This limited the highest possible BNS range in O2 to 40–60 $\mathrm {Mpc}$; the BNS range achieved was 25–30 $\mathrm {Mpc}$. Fused silica fibers will be reinstalled between O2 and O3. Other improvements such as reduction of technical noises, laser power increase and installation of a squeezed vacuum source will also be performed, bringing the AdV BNS range to 65–85 $\mathrm {Mpc}$ in 2018–2019. A configuration upgrade at this point will allow the range to increase to approximately 65–115 $\mathrm {Mpc}$ in 2020. The design sensitivity, with a BNS range of 125 $\mathrm {Mpc}$, is anticipated circa 2021. The corresponding BNS-optimized range would be 140 $\mathrm {Mpc}$. The sensitivity curves for the various AdV configurations are shown in Fig. 1.

The KAGRA detector (K1; Somiya 2012; Aso et al. 2013) is located at the Kamioka underground site. The first operation of a detector in an initial configuration with a simple Michelson interferometer occurred in March 2016 (Akutsu et al. 2018). The detector is now being upgraded to its baseline design configuration. Initial operation at room temperature is expected in 2018. Subsequently, the detector will be cryogenically cooled to reduce thermal noise. Early cryogenic observations may come in 2019–2020 with a range of 8–25 $\mathrm {Mpc}$. Since sensitivity will lag behind that of aLIGO and AdV, observing runs are planned to be short to allow commissioning to proceed as quickly as possible; longer observing runs may begin when the detector nears design sensitivity of 140 $\mathrm {Mpc}$. The exact timing of observations has yet to be decided, but it is currently intended to have a three-month observing run in early 2020, and a six-month run at the start of 2021. The sensitivity curves for the various KAGRA commissioning stages are shown in Fig. 1.

GEO 600 (Lück et al. 2010; Dooley et al. 2016) will continue to operate as a GW detector beyond O3 as techniques for improving the sensitivity at high frequency are investigated (Affeldt et al. 2014). At its current sensitivity, it is unlikely to contribute to detections, but with a deliberate focus on high frequency narrow band sensitivity at a few kilohertz, GEO 600 may contribute to the understanding of BNS merger physics, as well as sky localization for such systems, by around 2021. In the meantime, it will continue observing with frequent commissioning and instrument science investigations related to detuned signal recycling and novel applications of squeezed light, as well as increasing the circulating power and levels of applied squeezing (Abadie et al. 2011; Grote et al. 2013; Aasi et al. 2013a; Brown et al. 2017).

Finally, further upgrades to the LIGO and Virgo detectors, within their existing facilities (e.g., Hild et al. 2012; Miller et al. 2015; Aasi et al. 2015c) as well as future third-generation observatories, for example, the Einstein Telescope (Punturo et al. 2010; Hild et al. 2011; Sathyaprakash et al. 2012) or Cosmic Explorer (Abbott et al. 2017d), are envisioned in the future. It is also possible that for some sources, there could be multiband gravitational-wave observations, where detection with a space-borne detector like the *Laser Interferometer Space Antenna* (*LISA*; Amaro-Seoane et al. 2012, 2013) could provide early warning and sky localization (Sesana 2016), as well as additional information on system parameters (Vitale 2016), formation mechanisms (Nishizawa et al. 2016a, b; Breivik et al. 2016) and tests of general relativity (Barausse et al. 2016). These potential future developments are beyond the scope of this paper.

### Past and envisioned observing schedule

Keeping in mind the important caveats about commissioning affecting the scheduling and length of observing runs, the following are plausible scenarios for the operation of the ground-based GW detector network over the next decade:

- 2015–2016 (O1) A four-month run (12 September 2015–19 January 2016) with the two-detector H1L1 network at early aLIGO sensitivity (60–80$~\mathrm {Mpc}$ BNS range). This is now complete.
- 2016–2017 (O2) A nine-month run with H1L1, joined by V1 for the final month. O2 began on 30 November 2016, with AdV joining 1 August 2017 and ended on 25 August 2017. The expected aLIGO range was 80–120 $\mathrm {Mpc}$, and the achieved range was in the region of 60–100 $\mathrm {Mpc}$; the expected AdV range was 20–65 $\mathrm {Mpc}$, and the initial range was 25–30 $\mathrm {Mpc}$.
- 2018–2019 (O3) A year-long run with H1L1 at 120–170 $\mathrm {Mpc}$ and with V1 at 65–85 $\mathrm {Mpc}$ beginning about a year after the end of O2.
- 2020+ Three-detector network with H1L1 at full sensitivity of 190 $\mathrm {Mpc}$ and V1 at 65–115 $\mathrm {Mpc}$, later increasing to design sensitivity of 125 $\mathrm {Mpc}$.
- 2024+ H1L1V1K1I1 network at full sensitivity (aLIGO at 190 $\mathrm {Mpc}$, AdV at 125 $\mathrm {Mpc}$ and KAGRA at 140 $\mathrm {Mpc}$). Including more detectors improves sky localization (Klimenko et al. 2011; Veitch et al. 2012; Nissanke et al. 2013; Rodriguez et al. 2014; Pankow et al. 2018) as well as the fraction of coincident observational time. 2024 is the earliest time we imagine LIGO-India could be operational.

**Fig. 2:**
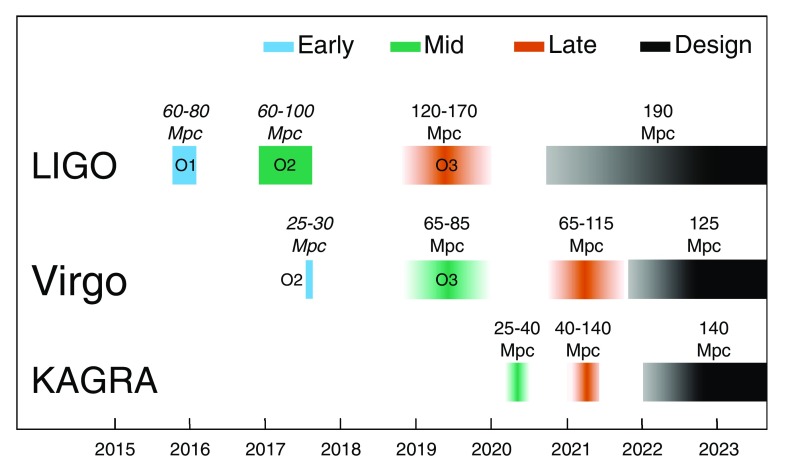
The planned sensitivity evolution and observing runs of the aLIGO, AdV and KAGRA detectors over the coming years. The colored bars show the observing runs, with the expected sensitivities given by the data in Fig. 1 for future runs, and the achieved sensitivities in O1 and in O2. There is significant uncertainty in the start and end times of planned the observing runs, especially for those further in the future, and these could move forward or backwards relative to what is shown above. The plan is summarised in Sect. 2.2

This timeline is summarized in Fig. 2; we do not include observing runs with LIGO-India yet, as these are still to be decided. Additionally, GEO 600 will continue observing, with frequent commissioning breaks, during this period. The observational implications of these scenarios are discussed in Sect. 4.

## Searches for gravitational-wave transients

Data from GW detectors are searched for many types of possible signals (Abbott et al. 2017j). Here we focus on signals from compact binary coalescence (CBCs) and on generic transient or *burst* signals. CBCs include BNS, neutron star–black hole (NS–BH) and BBH systems.

Observational results of searches for transient signals are reported in Abbott et al. (2016e, 2016c, 2016l, 2017b, 2016q, 2017l, 2017f, 2017g, 2017h, 2017i). The O1 results include two clear detections GW150914 (Abbott et al. 2016k) and GW151226 (Abbott et al. 2016g), and a lower significance candidate LVT151012 (Abbott et al. 2016e, c). All three originate from BBH coalescences (Abbott et al. 2016m, c). No other transient sources have been identified in O1 (Abbott et al. 2016q, 2017b, m). The first results of O2 have been announced: GW170104 (Abbott et al. 2017f), GW170608 (Abbott et al. 2017g) and GW170814 (Abbott et al. 2017h) are from BBH coalescences, and GW170817 (Abbott et al. 2017i) is the first detection of a BNS coalescence.

Using our observation of GW170817, we calculate that the merger rate density has a $90\%$ credible range of 320–$4740~\mathrm {Gpc^{-3}\,yr^{-1}}$ (Abbott et al. 2017i). This initial estimate assumes that neutron star masses are uniformly distributed between $1 M_\odot$ and $2 M_\odot$ and their dimensionless spins are less than 0.4. Compatible estimates for the merger rate were derived from the rate of electromagnetic transients similar to the counterpart of GW170817 (Siebert et al. 2017; Kasliwal et al. 2017; Smartt et al. 2017; Yang et al. 2017; Zhang et al. 2018). Complementary rate estimation based upon astrophysical population models and observations of Galactic BNS systems (e.g., Abadie et al. 2010b; Kim et al. 2013; Dominik et al. 2015; Vangioni et al. 2016; de Mink and Belczynski 2015; Eldridge et al. 2017; Belczynski et al. 2017; Kruckow et al. 2018) remains an active are of research.

While the rates per unit volume of NS–BH and BBH mergers are expected to be lower than for BNSs, the distance to which they can be observed is larger. Consequently, the predicted observable rates are comparable (Abadie et al. 2010b; Rodriguez et al. 2015; Abbott et al. 2016b; Li et al. 2017). From our observations of BBHs, we infer that their rate of mergers is in the range $\sim 1.2 \times 10^{-8}$–$2.13 \times 10^{-7}~\mathrm {Mpc^{-3}\,yr^{-1}}$ (Abbott et al. 2017f). The non-detection of NS–BHs in O1 allows us to place a $90\%$ upper limit of the merger rate of $3.6 \times 10^{-6}~\mathrm {Mpc^{-3}\,yr^{-1}}$, assuming $1.4 M_\odot + 5 M_\odot$ binaries with isotropically distributed spins (Abbott et al. 2016q); the upper limit on the rate decreases for higher mass black holes. Expected detection rates for other transient sources are lower and/or less well constrained.

For the purpose of detection, the gravitational waveform from the inspiral phase of a BNS coalescence is well modeled and matched filtering can be used to search for signals (Lindblom et al. 2008; Buonanno et al. 2009; Brown et al. 2012; Read et al. 2013; Abbott et al. 2016e; Harry et al. 2016). For systems containing black holes, or in which the component spin is significant, uncertainties in the waveform model can reduce the sensitivity of the search (Nitz et al. 2013; Harry et al. 2014; Dal Canton et al. 2015; Taracchini et al. 2014; Pan et al. 2014; Schmidt et al. 2015; Khan et al. 2016; Bustillo et al. 2017).

Searches for bursts make few assumptions on the signal morphology, using time–frequency decompositions to identify statistically significant excess-power transients in the data. Burst searches generally perform best for short-duration signals ($\lesssim 1~\mathrm {s}$), although search development remains an area of active research (e.g., Klimenko et al. 2008; Sutton et al. 2010; Chassande-Mottin et al. 2010; Thrane et al. 2011; Adams et al. 2013; Thrane and Coughlin 2013; Cornish and Littenberg 2015; Thrane et al. 2015; Kanner et al. 2016); their astrophysical targets include core-collapse supernovae, magnetar flares, BBH coalescences, cosmic string cusps, and, possibly, as-yet-unknown systems.

In the era of advanced detectors, we are searching in *near real-time* for CBC and burst signals for the purpose of rapidly identifying event candidates. A prompt notice of a potential GW transient can enable follow-up observations in the electromagnetic spectrum.

A first follow-up program including low-latency analysis, event candidate selection, position reconstruction and the sending of alerts to several observing partners (optical, X-ray, and radio) was implemented and exercised during the 2009–2010 LIGO–Virgo science run (Abadie et al. 2012c, b; Evans et al. 2012). Latencies of less than $1~\mathrm {h}$ were achieved.

In the present follow-up program, the LSC and Virgo distribute the times and sky localizations using the Gamma-ray Coordinates Network (GCN) system, widely used in the astronomical community for the multiwavelength follow-up of gamma-ray bursts.^3^ Messages are sent as machine-readable GCN Notices and as prose GCN Circulars, and astronomers communicate the results of observations using GCN Circulars. A shared infrastructure, including a database of results, allows observing partners to announce, coordinate and visualize the coverage of their observations.^4^

Prior to O1, 74 teams signed MOUs to participate in the electromagnetic follow-up program, and for the first event candidate later confirmed as GW150914, 63 were operational and covered the full electromagnetic spectrum (Abbott et al. 2016i, n). For the first observations with the advanced detectors, thorough checks were performed before alerts were released, resulting in latencies much greater than $1~\mathrm {h}$. We expect this latency to be improved in the future as we gain experience with the instruments, and aim for automatic alerts being sent out with only a few minutes latency; continued checks may lead to retractions of some of these low-latency alerts. In the case of GW150914, 25 teams responded to the GW alert and operated ground- and space-based instruments spanning 19 orders of magnitude in electromagnetic wavelength (Soares-Santos et al. 2016; Annis et al. 2016; Connaughton et al. 2016; Ackermann et al. 2016; Savchenko et al. 2016; Kasliwal et al. 2016; Palliyaguru et al. 2016; Hurley et al. 2016; Morokuma et al. 2016; Copperwheat et al. 2016; Lipunov et al. 2017a; Kawai et al. 2017; Smartt et al. 2016b, b; Evans et al. 2016c; Díaz et al. 2016; Brocato et al. 2017). Analyses of archival data were also performed (Troja et al. 2016; Tavani et al. 2016). No significant electromagnetic counterpart and no afterglow emission was found in optical, ultraviolet, X-rays, or $\mathrm {GeV}$ gamma rays. The weak transient found in *Fermi*-GBM data $0.4~\mathrm {s}$ after GW150914 (Connaughton et al. 2016; Bagoly et al. 2016) was not confirmed by other instruments like *INTEGRAL* SPI-ACS (Savchenko et al. 2016), *AGILE* (Tavani et al. 2016) or any other experiments of the InterPlanetary Network (Hurley et al. 2016). Models have been proposed to tentatively explain electromagnetic emission from BBHs, but there is no clear favorite as yet (Loeb 2016; Woosley 2016; Perna et al. 2016; Janiuk et al. 2017; Bartos et al. 2017; Stone et al. 2017; Li et al. 2016; Yamazaki et al. 2016; Ryan and MacFadyen 2017; Murase et al. 2016; Morsony et al. 2016; Dai et al. 2017; Lyutikov 2016; de Mink and King 2017). There was no significant neutrino emission temporally and spatially coincident with the event, and all detected neutrino candidates are consistent with the background (Adrian-Martinez et al. 2016; Gando et al. 2016; Aab et al. 2016; Abe et al. 2016; Agostini et al. 2017).

Similar follow-up campaigns have been performed for subsequent BBHs. LVT151012 was only identified in an offline search (Abbott et al. 2016e), as an online CBC search for BBHs was not running at the time. Nevertheless, some searching of archival data has been done, and no confident electromagnetic counterpart has been found (Racusin et al. 2017). GW151226 was identified by a low-latency online search (Abbott et al. 2016g) and a variety of teams followed up; no electromagnetic counterpart has been reported (Cowperthwaite et al. 2016; Smartt et al. 2016a; Copperwheat et al. 2016; Racusin et al. 2017; Evans et al. 2016b; Adriani et al. 2016; Palliyaguru et al. 2016; Yoshida et al. 2017; Serino et al. 2017; Brocato et al. 2017). No significant neutrino counterpart was found in coincidence with either LVT151012 or GW151226 (Albert et al. 2017c; Gando et al. 2016; Aab et al. 2016; Abe et al. 2016; Agostini et al. 2017). For GW170104 (Abbott et al. 2017f), a Circular with initial localization was sent in under $7~\mathrm {h}$; no confirmed electromagnetic or neutrino counterpart has been reported (Bhalerao et al. 2017; Verrecchia et al. 2017; Corsi et al. 2017; Stalder et al. 2017; Goldstein et al. 2017b; Agostini et al. 2017; Savchenko et al. 2017b; Albert et al. 2017a). Identification of GW170608 was delayed due to maintenance work being undertaken at Hanford at the time of the event (Abbott et al. 2017g), but a Circular was still issued within 14 h. No conclusive counterpart has yet been reported. GW170814 was the first event to be confidently detected by Virgo and the inclusion of the third detector significantly improved the localization for this event (Abbott et al. 2017h). A Circular and initial sky localization was issued in under 2 h. No counterpart has been reported so far (Arcavi et al. 2017b). A lack of counterparts is unsurprising given our current understanding of BBHs.

GW170817 was the first GW transient for which a firm electromagnetic counterpart was discovered (Abbott et al. 2017k). On 2017 August 17 12:41:06 UTC, *Fermi*-GBM triggered on a short GRB, GRB 170817A (Goldstein et al. 2017a), and a GCN was sent after $14~\mathrm {s}$. About $6~\mathrm {min}$ later, a GW trigger was identified; the signal was consistent with a BNS coalescence (Abbott et al. 2017i) occurring $\sim 1.7~\mathrm {s}$ before GRB 170817A (Abbott et al. 2017e), and a GCN was issued at 13:08:16 UTC. A three-detector GW localization was issued within $11~\mathrm {h}$ of detection. An extensive observing campaign was launched, leading to the discovery of the bright transient AT 2017gfo by the One-Meter, Two-Hemisphere team with the 1-$\mathrm {m}$ Swope Telescope (Coulter et al. 2017), and confirmed by other teams within an hour (Soares-Santos et al. 2017; Valenti et al. 2017; Arcavi et al. 2017a; Tanvir et al. 2017; Lipunov et al. 2017b). Subsequent infrared–ultraviolet observations targeted the transient and measured its brightness and spectrum, revealing a red-ward evolution (e.g., Villar et al. 2017). X-ray (Troja et al. 2017; Margutti et al. 2017; Haggard et al. 2017; Ruan et al. 2018; Pooley et al. 2017; D’Avanzo et al. 2018) and radio (Hallinan et al. 2017; Alexander et al. 2017; Mooley et al. 2018) afterglows were discovered at the position of the transient after $\sim 9~\mathrm {day}$ and $\sim 16~\mathrm {day}$ respectively; these were later joined by observation of the optical afterglow (Lyman et al. 2018; Margutti et al. 2018). Follow-up observations did not reveal any neutrino (Albert et al. 2017b) or high-energy gamma-ray (Abdalla et al. 2017) emission at the position of AT 2017gfo. These multimessenger observations support the hypothesis that GW170817 came from a BNS coalescence, which was the source of the short GRB 170817A (Goldstein et al. 2017a; Savchenko et al. 2017a) and a kilonova powered by the radioactive decay of r-process nuclei produced in the collision (Pian et al. 2017; McCully et al. 2017; Smartt et al. 2017; Chornock et al. 2017; Nicholl et al. 2017; Shappee et al. 2017).

The multimessenger observations for GW170817 allow for different studies ranging from astrophysics to fundamental physics and cosmology. The GW and gamma-ray data show that the BNS coalescence and the short gamma-ray are associated (Abbott et al. 2017e). The time delay of $\sim 1.7~\mathrm {s}$ between GW170817 and GRB 170817A places a constraint on the size and bulk Lorentz factor of the emitting region; furthermore, this delay constrains the difference between the speed of light and the speed of gravity, places new bounds on the violation of Lorentz invariance, and tests the equivalence principle by constraining the Shapiro delay between gravitational and electromagnetic radiation (Abbott et al. 2017e). These results limit the range of potential viable alternative theories of gravity (e.g., Creminelli and Vernizzi 2017; Sakstein and Jain 2017; Ezquiaga and Zumalacárregui 2017; Boran et al. 2018; Baker et al. 2017; Arai and Nishizawa 2017). GWs can be used as standard sirens for cosmological measurements (Schutz 1986). Combining the inferred GW distance with the redshift of the host galaxy NGC 4993, it was possible to infer the Hubble constant (Abbott et al. 2017a); the result is in agreement with the values determined from supernova (Riess et al. 2016) and cosmic microwave background measurements (Ade et al. 2016).

Increased detection confidence, improved sky localization, and identification of host galaxy and redshift are just some of the benefits of joint GW–electromagnetic observations. With this in mind, we focus on two points of particular relevance for follow-up of GW events: the source localization afforded by a GW network as well as the relationship between signal significance, or false alarm rate (FAR), and source localization.

### Detection and false alarm rates

Detection pipelines search the data looking for signal-like features. Candidate triggers flagged by a pipeline are assigned a detection statistic to quantify how signal-like they are. For CBC searches, this involves matching a bank of waveform templates (Sathyaprakash and Dhurandhar 1991; Owen 1996; Owen and Sathyaprakash 1999; Babak et al. 2006; Cokelaer 2007; Prix 2007; Harry et al. 2009; Ajith et al. 2014; Brown et al. 2012; Capano et al. 2016; Dal Canton and Harry 2017) to the data (Abbott et al. 2016e, c); for burst searches, requirements on waveform morphology are relaxed, but coherence of the signal in multiple detectors is required (Abbott et al. 2016l, 2017b). The detection statistic is used to rank candidates; we assess significance by comparing results with those from an estimated background distribution of noise triggers. It is difficult to theoretically model the behaviour of non-Gaussian noise, and therefore the distribution must be estimated from the data (Abadie et al. 2010a; Babak et al. 2013; Abadie et al. 2012a; Abbott et al. 2016a; Capano et al. 2017; Messick et al. 2017; Abbott et al. 2016e, c, l, 2017b; Nitz et al. 2017). From the background noise distribution we can map a value of the detection statistic to a FAR, the expected rate of triggers with detection statistics equal to or greater than that value, assuming that the data contain no signals. While each pipeline has its own detection statistic, they all compute a FAR, making it easy to compare results. The FAR, combined with the observation time, may then be used to calculate a *p* value, the probability of there being at least one noise trigger with a FAR this small or smaller in the observed time.^5^ As the FAR or *p* value of a trigger decreases, it becomes more significant, and more likely to be a genuine astrophysical signal.

The rate of noise triggers above a given SNR depends critically upon the data quality of the advanced detectors; non-stationary transients or *glitches* (Aasi et al. 2012, 2015b; Abbott et al. 2016d) produce an elevated background of loud triggers. Over 200,000 auxiliary channels record data on instrumental and environmental conditions (Effler et al. 2015; Abbott et al. 2016d). These channels act as witnesses of disturbances that may couple into the GW channel. An intensive study of the data quality is used to veto stretches of acquired data ranging from seconds to hours in duration. When a significant problem with the data is identified or a known instrumental issue affects the searches’ background, the contaminated data are removed from the analysis data set. Our experience to date is that this removes a small percentage of the data; for example, in O1 vetoes removed less than $5\%$ of the coincident data from the CBC analysis, with a single intermittent instrumental problem accounting for $4.65\%$ of that total (Abbott et al. 2016d, c, 2017b, 2018). For low-mass CBC searches, the waveforms are well modeled, and signal consistency tests reduce the background significantly (Allen 2005; Cannon et al. 2015; Usman et al. 2016). For burst sources which are not well modeled, or which spend only a short time in the detectors’ sensitive band, it is more difficult to distinguish between the signal and a glitch, and so a reduction of the FAR comes at a higher cost in terms of reduced detection efficiency (or live-time if more vetoes are used).

**Fig. 3:**
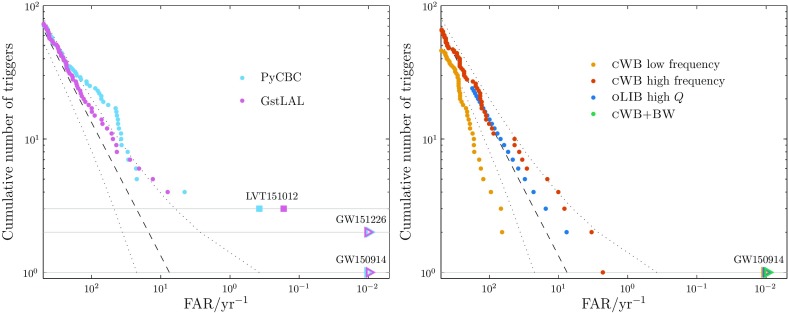
Offline transient search results for the first observing run: the cumulative number of triggers with false alarm rates (FARs) smaller than the abscissa value. The dashed line shows the expected noise-only distribution, and the dotted lines show the $90\%$ confidence interval assuming no signals. Potential signals are identified by having smaller FARs than expected. The plots are truncated at a minimum FAR of $10^{-2}~\mathrm {yr^{-1}}$. *Left*: Compact binary coalescence search results (Abbott et al. 2016c, q). We show results from two search algorithms, GstLAL (Cannon et al. 2012; Privitera et al. 2014; Messick et al. 2017) and PyCBC (Dal Canton et al. 2014; Usman et al. 2016). The most significant triggers for both are LVT151012, GW151226 and GW150914; GW150914 and GW151226 have FARs less than $10^{-2}~\mathrm {yr^{-1}}$. *Right*: Burst search results (Abbott et al. 2017b). We show results from three search algorithms, coherent Wave Burst (cWB; Klimenko et al. 2016, 2008), Omicron–LALInferenceBurst (oLIB; Lynch et al. 2017) and BayesWave follow-up of cWB (cWB+BW; Kanner et al. 2016). All three found GW150914 (the only cWB trigger above the BayesWave follow-up threshold) with a FAR less than $10^{-2}~\mathrm {yr^{-1}}$. GW151226 and LVT151012 fall below the burst search’s detection threshold

Search pipelines are run both online, analysing data as soon as they are available in order to provide low-latency alerts of interesting triggers, and offline, taking advantage of improved calibration of the data and additional information regarding data quality. In Fig. 3, we show the offline transient search results for O1.^6^

For CBC, we show the cumulative number of triggers at a given FAR for two pipelines: PyCBC (Dal Canton et al. 2014; Usman et al. 2016) and GstLAL (Cannon et al. 2012; Privitera et al. 2014; Messick et al. 2017). In the O2 analysis, PyCBC uses an improved detection statistic (Nitz et al. 2017). GW150914, LVT151012 and GW151226 are visible in both the GstLAL and PyCBC results (Abbott et al. 2016e, c) shown in Fig. 3.

For bursts, we show distributions for coherent Wave Burst (cWB; Klimenko et al. 2016, 2008), Omicron–LALInferenceBurst (oLIB; Lynch et al. 2017) and BayesWave (Cornish and Littenberg 2015; Littenberg and Cornish 2015). The cWB analysis is split into two frequency bands, above and below $1024~\mathrm {Hz}$. The oLIB search is split into two bins, based upon the quality factor *Q* of the sine–Gaussian it uses to model the signal; no triggers were identified by the low-*Q* search. BayesWave is run as a follow-up to triggers identified by cWB (Kanner et al. 2016), and hence is not completely independent. GW150914 was identified by all three search algorithms (Abbott et al. 2016l, 2017b).

For CBC signals, we conservatively estimate that a network SNR threshold of $\rho _{c} \simeq 12$ is required for a FAR below $\sim 10^{-2}~\mathrm {yr}^{-1}$ in the advanced-detector era (Abadie et al. 2012f; Aasi et al. 2016; Berry et al. 2015). A combined SNR of 12 corresponds to a single-detector SNR of 8.5 in each of two detectors, or 7 in each of three detectors (assuming an orientation and sky location for which the detectors have equal sensitivity). The exact threshold will depend upon data quality in each observing run as well as the mass of the source; in O1, we found that the threshold SNR was lower, around 10.

### Localization

Following the detection of a GW transient posterior probability distributions for the position are constructed following a Bayesian framework (Veitch et al. 2015; Cornish and Littenberg 2015; Singer and Price 2016; Abbott et al. 2016m), with information for the sky localization coming from the time of arrival, plus the phase and amplitude of the GW.

An intuitive understanding of localization can be gained by considering triangulation using the observed time delays between sites (Fairhurst 2009, 2011). The effective single-site timing accuracy is approximately

1 $$\begin{aligned} \sigma _t = \frac{1}{2\pi \rho \sigma _f} \, , \end{aligned}$$

where $\rho$ is the SNR in the given detector and $\sigma _f$ is the effective bandwidth of the signal in the detector, typically of order $100~\mathrm {Hz}$. Thus a typical timing accuracy is on the order of $10^{-4}~\mathrm {s}$ (about 1 / 100 of the $10~\mathrm {ms}$ light travel time between sites). This sets the localization scale. The simple model of Eq. (1) ignores many other relevant issues such as information from the signal amplitudes and phases across the detector network, uncertainty in the emitted gravitational waveform, instrumental calibration accuracies, and correlation of sky location with other binary parameters (Fairhurst 2009; Vitale and Zanolin 2011; Vitale et al. 2012; Nissanke et al. 2011; Veitch et al. 2012; Nissanke et al. 2013; Singer et al. 2014; Berry et al. 2015; Singer and Price 2016; Fairhurst 2017). While many of these affect the measurement of the time of arrival in individual detectors, such factors are largely common between two similar detectors, so the time difference between the two detectors is relatively uncorrelated with these additional parameters.

The timing-triangulation approach underestimates how well a source can be localized, since it does not include all the relevant information. Its predictions can be improved by introducing the requirement of phase and amplitude consistency between detectors (Grover et al. 2014; Fairhurst 2017): it always performs poorly for a two-detector network, but, with the inclusion of phase coherence, can provide an estimate for the average performance of a three-detector network (Berry et al. 2015).

**Fig. 4:**
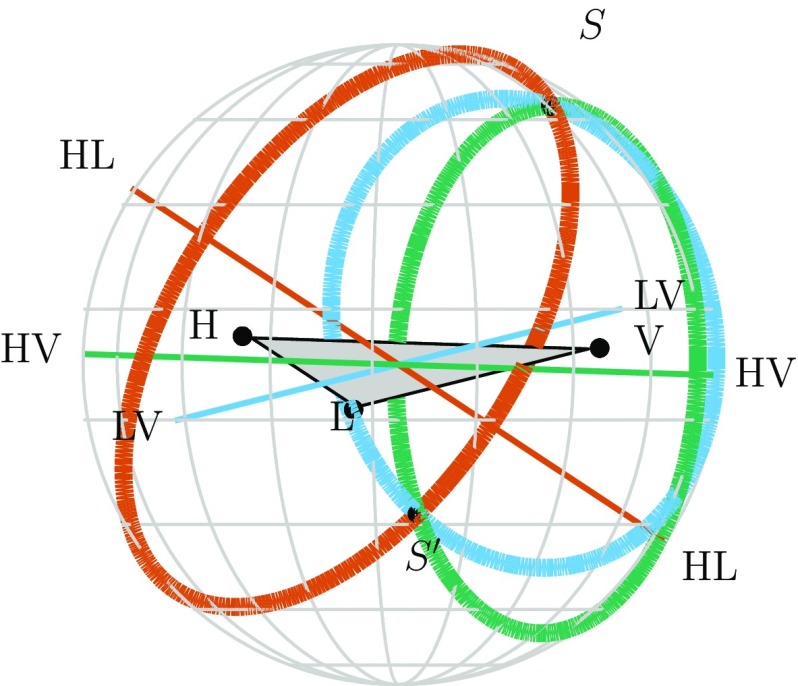
Source localization by timing triangulation for the aLIGO–AdV network. The locations of the three detectors are indicated by black dots, with LIGO Hanford labeled H, LIGO Livingston as L, and Virgo as V. The locus of constant time delay (with associated timing uncertainty) between two detectors forms an annulus on the sky concentric about the baseline between the two sites (labeled by the two detectors). For three detectors, these annuli may intersect in two locations. One is centered on the true source direction (*S*), while the other ($S^{\prime }$) is its mirror image with respect to the geometrical plane passing through the three sites. For four or more detectors there is a unique intersection region of all of the annuli. Image adapted from Chatterji et al. (2006)

**Fig. 5:**
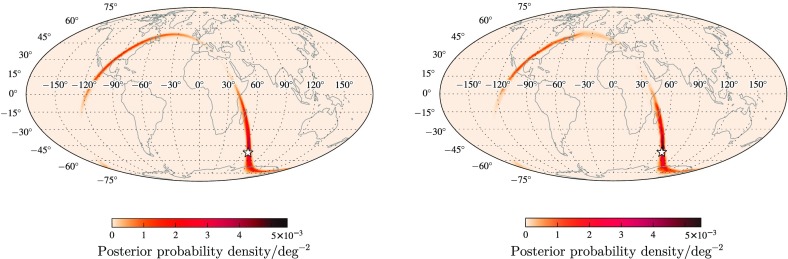
Posterior probability density for sky location for an example simulated binary neutron star coalescence observed with a two-detector network. *Left*: Localization produced by the low-latency bayestar code (Singer et al. 2014; Singer and Price 2016). *Right*: Localization produced by the higher-latency (neglecting spin) LALInference (Veitch et al. 2015), which also produces posterior estimates for other parameters. These algorithms are discussed in Sect. 3.2.1, and agreement between them shows that the low-latency localization is comparable to the one produced by the higher-latency pipelines. The star indicates the true source location. The source is at a distance of $266~\mathrm {Mpc}$ and has a network signal-to-noise ratio of $\rho _{c} = 13.2$ using a noise curve appropriate for the first aLIGO run (O1, see Sect. 4.1). The plot is a Mollweide projection in geographic coordinates. Figure reproduced with permission from Berry et al. (2015), copyright by AAS; further mock sky localizations for the first two observing runs can be found at www.ligo.org/scientists/first2years/ for binary neutron star signals and www.ligo.org/scientists/burst-first2years/ for burst signals

Source localization using only timing for a two-site network yields an annulus on the sky; see Fig. 4. Additional information such as signal amplitude and phase, and precession effects resolve this to only parts of the annulus, but even then sources will only be localized to regions of hundreds to thousands of square degrees (Singer et al. 2014; Berry et al. 2015). An example of a two-detector BNS localization is shown in Fig. 5. The posterior probability distribution is primarily distributed along a ring, but this ring is broken, such that there are clear maxima.

For three detectors, the time delays restrict the source to two sky regions which are mirror images with respect to the plane passing through the three sites. It is often possible to eliminate one of these regions by requiring consistent amplitudes in all detectors (Fairhurst 2017). For signals just above the detection threshold, this typically yields regions with areas of several tens to hundreds of square degrees. If there is significant difference in sensitivity between detectors, the source is less well localized and we may be left with the majority of the annulus on the sky determined by the two most sensitive detectors. With four or more detectors, timing information alone is sufficient to localize to a single sky region, and the additional baselines help to limit the region to under $10~\mathrm {deg^2}$ for some signals.

From Eq. (1), it follows that the *linear* size of the localization ellipse scales inversely with the SNR of the signal and the frequency bandwidth of the signal in the detector (Berry et al. 2015). For GWs that sweep across the band of the detector, such as CBC signals, the effective bandwidth is $\sim 100~\mathrm {Hz}$, determined by the most sensitive frequencies of the detector. Higher mass CBC systems merge at lower frequencies and so have a smaller effective bandwidth. For burst transients, the bandwidth $\sigma _f$ depends on the specific signal. For example, GWs emitted by various processes in core-collapse supernovae are anticipated to have relatively large bandwidths, between 150 Hz and $500~\mathrm {Hz}$ (Dimmelmeier et al. 2008; Ott 2009; Yakunin et al. 2010; Ott et al. 2011), largely independent of detector configuration. By contrast, the sky localization region for narrowband burst signals may consist of multiple disconnected regions and exhibit fringing features; see, for example, Klimenko et al. (2011), Abadie et al. (2012c), Essick et al. (2015).

In addition to localizing sources on the sky, for CBC signals it is possible to provide distance estimates, since the waveform amplitude is inversely proportional to the luminosity distance (Veitch et al. 2015; Abbott et al. 2016m). Uncertainty in distance measurement is dominated by the degeneracy with the binary’s inclination, which also determines the signal amplitude (Cutler and Flanagan 1994; Nissanke et al. 2010; Aasi et al. 2013c). The degeneracy could be broken by observing with more non co-aligned detectors (Veitch et al. 2012; Rodriguez et al. 2014), or if precession of the orbital plane is observed (Vecchio 2004; van der Sluys et al. 2008; Vitale et al. 2014), but this is not expected for slowly spinning BNS (Farr et al. 2016). Distance information can further aid the hunt for counterparts, particularly if the the localization can be used together with galaxy catalogs (Nissanke et al. 2013; Hanna et al. 2014; Fan et al. 2014; Blackburn et al. 2015; Singer et al. 2016a).

Some GW searches are triggered by electromagnetic observations, and in these cases some localization information is known *a priori*. For example, in GW searches triggered by gamma-ray bursts (Abadie et al. 2012e; Aasi et al. 2014c, b; Abbott et al. 2017l), the triggering space-based telescope provides a localization. The coincident observation of GW170817 (Abbott et al. 2017k) and GRB 170817A (Goldstein et al. 2017a; Savchenko et al. 2017a) confirms that BNS mergers are a progenitor for short gamma-ray bursts (Abbott et al. 2017d), and therefore that gamma-ray bursts are interesting targets for triggered GW searches. Other possible targets for these externally-triggered GW searches could be electromagnetic or neutrino emission from Galactic core-collapse supernovae. It is therefore of great scientific value to have telescopes capable of observing the high-energy spectrum operating during the advanced-detector era (and beyond). Furthermore, the rapid identification of a GW counterpart to such a trigger could prompt further spectroscopic studies and longer, deeper follow-up in different wavelengths that may not always be done in response to gamma-ray bursts (cf. Abbott et al. 2017k). This is particularly important for gamma-ray bursts with larger sky localization uncertainties, such as those reported by the *Fermi*-GBM, which are not followed up as frequently as bursts reported by *Swift* or *Fermi*-LAT; in the case of GW170817, the LIGO–Virgo localization was tighter than the localization from *Fermi*-GBM and *INTEGRAL* (Abbott et al. 2017e; Goldstein et al. 2017a; Savchenko et al. 2017a) and also showed that the source was nearby ($40^{+8}_{-14}~\mathrm {Mpc}$; Abbott et al. 2017i), making it a prime target for further follow-up. All GW data are stored permanently, so that it is possible to perform retroactive analyses at any time.

#### Localization of binary neutron star coalescences

Providing prompt localizations for GW signals helps to maximise the chance that electromagnetic observatories can catch a counterpart. Localizations are produced at several different latencies, with updates coming from more computationally expensive algorithms that refine our understanding of the source.

For CBC signals, rapid localization is performed using bayestar (Singer and Price 2016), a Bayesian parameter-estimation code that computes source location using output from the detection pipeline. It can produce sky localizations (as in Fig. 5) with latencies of only a few seconds. A similar approach to low-latency localization has been separately developed by Chen and Holz (2015), which find results consistent with bayestar. bayestar can also provide distance estimates (Singer et al. 2016a). These can be easily communicated as an additional component of the sky localization: for each line of sight, the distance posterior probability is approximated as a Gaussian multiplied by the distance squared (Singer et al. 2016a, b).^7^ Results from bayestar are shared at low latency for prompt follow-up efforts.

At higher latency, CBC parameter estimation is performed using the stochastic sampling algorithms of LALInference (Veitch et al. 2015). LALInference constructs posterior probability distributions for system parameters, not just location like bayestar, but also mass, orientation, etc. (Aasi et al. 2013c; Abbott et al. 2016m), by matching GW templates to the detector strain (Cutler and Flanagan 1994; Jaranowski and Królak 2012). Computing these waveforms is computationally expensive; this expense increases as the detectors’ low-frequency sensitivity improves and waveforms must be computed down to lower frequencies. The quickest LALInference BNS follow-up is computed using waveforms that do not include the full effects of component spins (Singer et al. 2014; Berry et al. 2015; Abbott et al. 2017f), localizations can then be reported with latency of hours to a couple of days. Parameter estimation is then performed using more accurate waveform approximates, those that include fuller effects of spin precession and the effects of tidal distortions of neutron stars (Farr et al. 2016; Abbott et al. 2016h, 2017f, i). Provided that BNSs are slowly spinning (Mandel and O’Shaughnessy 2010), the restrictions on the spins should cause negligible difference between the mid-latency LALInference and the high-latency fully spinning LALInference localizations (Farr et al. 2016). Methods of reducing the computational cost are actively being investigated (e.g., Canizares et al. 2013; Pürrer 2014; Canizares et al. 2015; Smith et al. 2016; Vinciguerra et al. 2017).

**Fig. 6:**
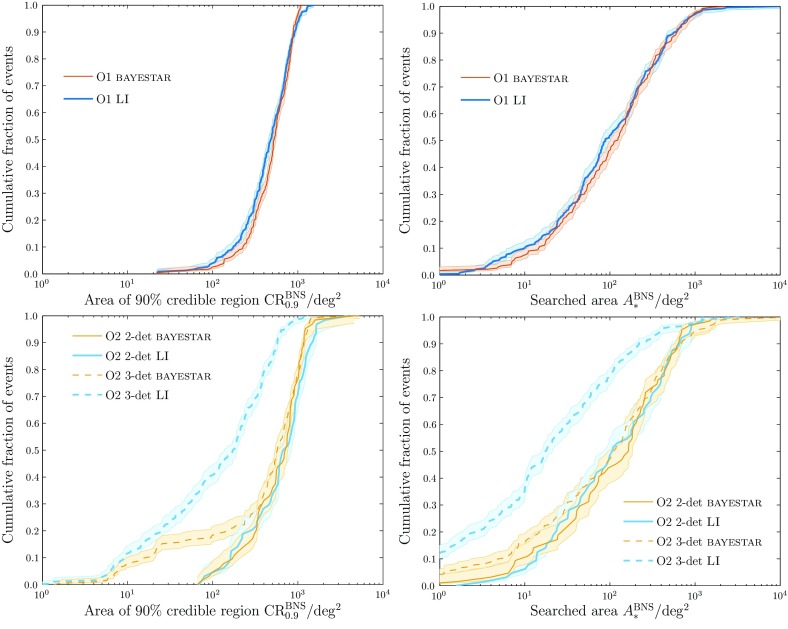
Anticipated binary neutron star sky localization during the first two observing runs (*top*: O1, see Sect. 4.1; *bottom*: O2, see Sect. 4.2). Detector sensitivities were taken to be in the middle of the early (for aLIGO in O1 and AdV in O2) and mid (for aLIGO in O2) bands of Fig. 1. The plots show the cumulative fractions of events with sky-localization areas smaller than the abscissa value. *Left*: Sky area of $90\%$ credible region $\mathrm {CR}_{0.9}^\mathrm {BNS}$, the (smallest) area enclosing $90\%$ of the total posterior probability. *Right*: Searched area $A_*^\mathrm {BNS}$, the area of the smallest credible region containing the true position. Results are shown for the low-latency bayestar (Singer and Price 2016) and higher-latency (neglecting spin) LALInference (LI; Veitch et al. 2015) codes. The O2 results are divided into those where two detectors (2-det) are operating in coincidence, and those where three detectors (3-det) are operating: assuming a duty cycle of 70–$75\%$ for each instrument, the two-detector network would be operating for 42–$44\%$ of the total time and the three-detector network 34–$42\%$ of the time. The shaded areas indicate the $68\%$ confidence intervals on the cumulative distributions. A detection threshold of a signal-to-noise ratio of 12 is used and results are taken from Berry et al. (2015), Singer et al. (2014)

Sky localization results from an astrophysically motivated population of BNS signals, assuming a detection threshold of a SNR of 12, are shown in Fig. 6 (Singer et al. 2014; Berry et al. 2015). Results are quantified using the $90\%$ credible region $\mathrm {CR}_{0.9}$, the smallest area enclosing $90\%$ of the total posterior probability, and the searched area $A_*$, the area of the smallest credible region that encompasses the true position (Sidery et al. 2014): $\mathrm {CR}_{0.9}$ gives the area of the sky that must be covered to expect a $90\%$ chance of including the source location, and $A_*$ gives the area that would be viewed before the true location is found using the given sky localization. Results from both the low-latency bayestar and mid-latency LALInference analyses are shown. These are discussed further in Sects. 4.1 and 4.2. The two-detector localizations are slightly poorer in O2 than in O1. This is because although the detectors improve in sensitivity at every frequency, with the assumed noise curves the BNS signal bandwidth is lower in O2 for a given SNR because of enhanced sensitivity at low frequencies (Singer et al. 2014). Sky localization improves with the expansion of the detector network (Schutz 2011; Klimenko et al. 2011; Veitch et al. 2012; Rodriguez et al. 2014; Gaebel and Veitch 2017; Pankow et al. 2018).

The results in Fig. 6 show there is negligible difference between the low-latency bayestar and the LALInference analyses if the BNS signal is loud enough to produce a trigger in all detectors. However, when the signal is sub-threshold in one, LALInference could give a more precise localization, as it still uses strain data from the non-triggered detector (Singer et al. 2014; Singer and Price 2016). In preparation for the start of joint three-detector observations in O2, the online CBC pipelines and bayestar have been enhanced to capture and make use of sub-threshold signals in all detectors. Consequently, there should be negligible difference between the low-latency bayestar and LALInference localizations even for events that register weakly in one or more detectors. This was the case for GW170817, where both analyses produced a $90\%$ credible area of $\sim 30~\mathrm {deg^2}$ (Abbott et al. 2017k).

For the BNS signals from the sky-localization studies, the average fractional distance uncertainty, defined as the posterior standard deviation divided by the mean, is $\sim 0.25$–0.30 (Berry et al. 2015; Farr et al. 2016).

LALInference also has the ability to include the effects of the detectors’ calibration uncertainty on parameter estimation (Abbott et al. 2016m, c). Calibration is refined as additional measurements are taken, hence sky localization can improve as uncertainty is reduced. Initial results for GW150914 assumed a calibration uncertainty of $10\%$ for the amplitude of the GW strain and $10~\mathrm {deg}$ for its phase (Abbott et al. 2017c). Incorporating this calibration uncertainty into the analysis, the $90\%$ credible area was $610~\mathrm {deg^2}$ (Abbott et al. 2016m). By the end of O1, the calibration uncertainty had been improved, such that the $90\%$ credible area was $230~\mathrm {deg^2}$ (Abbott et al. 2016c). If the detectors were assumed to be perfectly calibrated, such that calibration uncertainty could be ignored, the $90\%$ credible area would be $150~\mathrm {deg^2}$. Sky localization is particularly sensitive to calibration uncertainty and distance is less affected. For GW150914, the initial distance estimate was $410_{-180}^{+160}~\mathrm {Mpc}$ (Abbott et al. 2016m), the estimate at the end of the run was $420_{-180}^{+150}~\mathrm {Mpc}$, and the equivalent result without calibration uncertainty was $420_{-170}^{+140}~\mathrm {Mpc}$ (Abbott et al. 2016c). The effects of calibration uncertainty depend upon the signal’s SNR, bandwidth and position of the source relative to the detectors. For GW151226, LVT151012 and GW170104, there is negligible difference between the sky areas or distances with and without calibration uncertainty using the final calibration uncertainties (Abbott et al. 2016c, 2017f).

#### Localization of bursts

Sky localizations are also produced for burst triggers and distributed for follow up. The lowest latency burst sky localizations are produced as part of the cWB detection pipeline (Klimenko et al. 2008, 2016). Sky localizations are produced using a constrained likelihood algorithm that coherently combines data from all the detectors. The cWB sky localizations are calculated with a latency of a few minutes; following detection, further parameter-estimation codes analyze the data.

At higher latency, burst signals are analyzed by LALInferenceBurst (LIB), a stochastic sampling algorithm similar to the LALInference code used to reconstruct CBC signals (Veitch et al. 2015), and BayesWave, a reversible jump Markov-chain Monte Carlo algorithm that models both signals and glitches (Cornish and Littenberg 2015). LIB uses sine–Gaussian waveforms (in place of the CBC templates used by LALInference), and can produce sky localizations in a few hours. BayesWave uses a variable number of sine–Gaussian wavelets to model a signal and glitches while also fitting for the noise spectrum using BayesLine (Littenberg and Cornish 2015); it produces sky localizations with a latency of a few days.

**Fig. 7:**
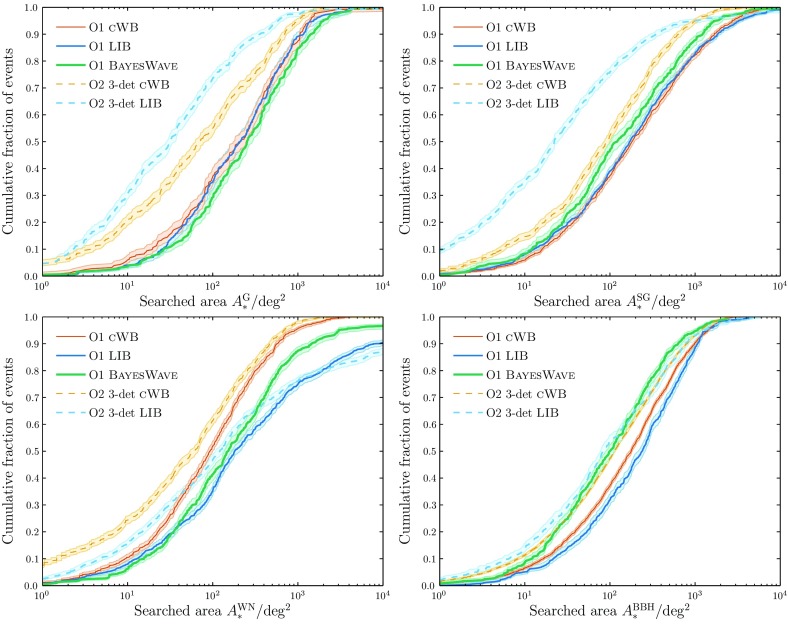
Simulated sky localization for Gaussian (G; *top left*), sine–Gaussian (SG; *top right*), broadband white-noise (WN; *bottom left*) and binary black hole (BBH; *bottom right*) bursts during the first two observing runs (O1, see Sect. 4.1, and O2, see Sect. 4.2). The plots show the cumulative fractions of events with searched areas $A_*$ smaller than the abscissa value. Results are shown for the low-latency coherent WaveBurst (CWB; Klimenko et al. 2005, 2008, 2016), and higher-latency LALInferenceBurst (LIB; Veitch et al. 2015) and BayesWave (Cornish and Littenberg 2015) codes. The O2 results consider only a three-detector (3-det) network; assuming an instrument duty cycle of 70–$75\%$, this would be operational 34–$42\%$ of the time. The BayesWave results are only for O1 and include only events that could be detected by the code. The shaded areas indicate the $68\%$ confidence intervals on the cumulative distributions. A detection threshold of a false alarm rate of approximately $1~\mathrm {yr^{-1}}$ is used for cWB and LIB, and BayesWave is run as a follow-up for cWB triggers. Results are taken from Essick et al. (2015) and Bécsy et al. (2016)

The sky-localization performance of burst algorithms depends upon the type of signal. Studies of burst localization using BayesWave in the first year of the advanced-detector era, and using cWB and LIB in the first two years have been completed in Bécsy et al. (2016) and Essick et al. (2015), respectively. These assumed sensitivities in the middle of the early band for aLIGO in O1, and in the middle of the mid band for aLIGO and the early band for AdV in O2. Sky localization was quantified by the searched area. For the cWB and LIB pipelines an approximate FAR threshold of $1~\mathrm {yr^{-1}}$ was used to select events; BayesWave was run as a follow-up to triggers identified by cWB (Kanner et al. 2016), and sky localization was only performed on triggers also detected by BayesWave (and not classified as noise or a glitch). The median localization was shown to be $\sim 100$–$200~\mathrm {deg^2}$ for a two-detector network in O1 and $\sim 60$–$110~\mathrm {deg^2}$ for a three-detector network in O2, with the localization and relative performance of the algorithms depending upon the waveform morphology. Results for Gaussian, sine–Gaussian, broadband white-noise and BBH waveforms are shown in Fig. 7 (for the two-detector O1 network and the three-detector O2 network, cf. Fig. 6). The variety of waveform morphologies reflect the range of waveforms that could be detected in a burst search (Abadie et al. 2012c).

A high mass BBH, like GW150914 (Abbott et al. 2016k), could be detected by both burst and CBC analyses. In this case, we expect that the CBC localization, which makes use of the additional information available from constraining signals to match waveform templates, is more accurate than the burst localization (cf. Vitale et al. 2016).

## Observing scenarios

In this section we estimate the sensitivity, possible number of detections, and localization capability for each of the observing runs laid out in Sect. 2.2. We discuss each future observing run in turn and also summarize the results in Table 3.

In the following, we estimate the expected number of BNS coalescence detections using the inferred $90\%$ credible range for the BNS source rate density, 320–$4740~\mathrm {Gpc^{-3}\,yr^{-1}}$ (Abbott et al. 2017i). Given the detectors’ noise spectral densities, the $\rho _c$ detection threshold can be converted into the (source sky-location and orientation averaged) BNS sensitive detection range $R_\mathrm {BNS}$ (Abadie et al. 2010b, 2012g). From this, the BNS source rate density can be converted into an estimate of the number of expected detected events; this estimate carries the large error on the source rate density. Similar estimates may be made for NS–BH binaries using the fact that the NS–BH range is approximately a factor of 1.6 larger than the BNS range,^8^ though the uncertainty in the NS–BH source rate density is slightly larger (Abadie et al. 2010b). We assume a nominal $\rho _{c}$ threshold of 12, at which the expected FAR is $\sim 10^{-2}~\mathrm {yr}^{-1}$. However, such a stringent threshold may not be appropriate for selecting candidate triggers for electromagnetic follow-up. For example, selecting CBC candidates at thresholds corresponding to a higher background rate of $1~\mathrm {yr}^{-1}$ ($100~\mathrm {yr}^{-1}$) would increase the number of true signals subject to electromagnetic follow-up by about $30\%$ ($90\%$). The area localization for these low-threshold signals is, on average, only fractionally worse than for the high-threshold population—by approximately $20\%$ ($60\%$). The localization of NS–BH signals is expected to be similar to that of BNS signals.

**Table 2:** Percentage of time during the first observing run that the LIGO detectors spent in different operating modes as entered by the on-duty operator

Detector		Hanford	Livingston
Operating mode	Observing	64.6%	57.4%
	Locking	17.9%	16.1%
	Environmental	9.7%	19.8%
	Maintenance	4.4%	4.9%
	Commissioning	2.9%	1.6%
	Planned engineering	0.1%	0.0%
	Other	0.4%	0.4%

Since several factors may influence detector operation at any given time, there is a certain subjectivity to the assignments. Maintenance includes a planned 4-h weekly period ($\sim 2.4\%$ of the total). Coincident operation of the detectors occurred $\sim 43\%$ of the time

For typical burst sources, the gravitational waveform is not well known. However, the performance of burst searches is largely independent of the detailed waveform morphology (Abadie et al. 2012a; Essick et al. 2015), allowing us to quote an approximate sensitive range determined by the total energy $E_\mathrm {GW}$ emitted in GWs, the central frequency $f_0$ of the burst, the detector noise spectrum *S*(*f*), and the single-detector SNR threshold $\rho _\mathrm {det}$ (Sutton 2013),

2 $$\begin{aligned} R_\mathrm {burst} \simeq \left[ \frac{G}{2\pi ^2c^3}\frac{E_\mathrm {GW}}{S(f_0) f_0^2 \rho _\mathrm {det}^2}\right] ^{1/2} \, . \end{aligned}$$

In this article, we quote ranges using $E_\mathrm {GW}=10^{-2}\,M_\odot c^2$ and $f_0=150~\mathrm {Hz}$; $E_\mathrm {GW}=10^{-2}\,M_\odot c^2$ is an optimistic value for GW emission from stellar collapse (e.g., Abadie et al. 2012e); the uncertainty in $E_\mathrm {GW}$ means that the quoted burst ranges are more uncertain than their BNS counterparts. We use a single-detector SNR threshold of 8, corresponding to a typical network SNR of $\sim 12$.

The run durations discussed below are in calendar time. In O1, the H1–L1 network had a duty factor of approximately $43\%$. Table 2 illustrates how the up time for each detector was impacted by various activities or the environment. The two biggest non-observing categories for each detector are Locking and Environmental. Locking refers to the amount of time spent in bringing the interferometers from an uncontrolled state to their lowest noise configuration (Staley et al. 2014). Environmental effects include earthquakes, wind and the microseism noise arising from ocean storms (Effler et al. 2015; Abbott et al. 2016d). The latter two effects have seasonal variation, with the prevalence of storms being higher during the winter months. L1 has a greater sensitivity to microseism noise and to earthquakes than H1 mainly due to the local geophysical environment (Daw et al. 2004). During O1, L1 lost over twice as much observing time to earthquakes, microseism noise and wind than did H1. While we can expect some improvement in duty factors from operating during non-winter months, we can continue to expect at least a $10\%$ impact on operating time from environmental effects. Adding in maintenance, both planned and unplanned, and time spent in locking we currently expect duty factors of at most 70–$75\%$ for each instrument during extended runs. Assuming downtime periods are uncorrelated among detectors, this means that all detectors in a three-detector network will be operating in coincidence approximately 34–$42\%$ of the time, and at least two detectors will be operating for 78–$84\%$ of the time. For a four-detector network, three or more detectors will be operational around 65–$74\%$ of the time, and for a five-detector network, three of more detectors will be operating for 84–$90\%$ of the time. Our estimates for the expected number of detections and the fraction of sources localized account for these duty cycles. The downtime periods are sometimes correlated between detectors, for example, planned maintenance periods are often coordinated, and so these coincidence times may be conservative estimates. The number of detections also account for the uncertainty in the detector sensitive ranges as indicated in Fig. 1, but do not include any cosmological evolution of the merger rate.

### 2015–2016 run (O1): aLIGO

This was the first advanced-detector observing run, lasting four months, starting 12 September 2015 and ending 19 January 2016.

The aLIGO sensitivity was expected to be similar to the early band in Fig. 1, with a BNS range of 40–80 $\mathrm {Mpc}$, and a burst range of 40–$60~\mathrm {Mpc}$ for $E_\mathrm {GW}=10^{-2}\,M_\odot c^2$. The achieved sensitivity was at the better end of this span, with a BNS range of $\sim$ 60–80 $\mathrm {Mpc}$.

The O1 BNS search volume was $\sim 2 \times 10^5~\mathrm {Mpc^3\,yr}$, and the dominant source of uncertainty on this value is the calibration of the detectors (Abbott et al. 2016q). The search volume is $V_z T$, where $V_z = (4\pi /3) R^3$ is the time-averaged volume surveyed and *T* is the observing time incorporating the effects of the detectors’ duty cycles. We would therefore expect 0.05–1 BNS detections. No BNS detections were made, consistent with these expectations (Abbott et al. 2016q).

With the two-detector H1–L1 network any detected events are unlikely to be well localized. A full parameter-estimation study using realistic detector noise and an astrophysically-motivated source catalog has been completed for 2015–2016 (Berry et al. 2015).^9^ This used a noise curve in the middle of the early range shown in Fig. 1—the early curve specified in Barsotti and Fritschel (2012). The distribution of results is shown in Fig. 6. In Table 3, we present results calculated using bayestar (Singer and Price 2016) for a population of BNS signals, assuming an SNR threshold of 12; the results agree with those of Berry et al. (2015). The median $90\%$ credible region for is 460–530 $\mathrm {deg}^2$; the searched area $A_*^\mathrm {BNS}$ is smaller than $20~\mathrm {deg}^2$ for 14–$17\%$ of events and smaller than $5~\mathrm {deg}^2$ in 4–$6\%$.

**Table 3:** Summary of a plausible observing schedule, expected sensitivities, and source localization with the Advanced LIGO, Advanced Virgo and KAGRA detectors, which will be strongly dependent on the detectors’ commissioning progress

Epoch			2015 – 2016	2016 – 2017	2018 – 2019	2020+	2024+
Planned run duration			$4~\mathrm {months}$	$9~\mathrm {months}$	$12~\mathrm {months}$	(per year)	(per year)
Expected burst range/$\mathrm {Mpc}$		LIGO	40 – 60	60–75	75–90	105	105
		Virgo	–	20–40	40–50	40–70	80
		KAGRA	–	–	–	–	100
Expected BNS range/$\mathrm {Mpc}$		LIGO	40–80	80–120	120–170	190	190
		Virgo	–	20–65	65–85	65–115	125
		KAGRA	–	–	–	–	140
Achieved BNS range/$\mathrm {Mpc}$		LIGO	60–80	60–100	–	–	–
		Virgo	–	25–30	–	–	–
		KAGRA	–	–	–	–	–
Estimated BNS detections			0.05–1	0.2–4.5	1–50	4–80	11–180
Actual BNS detections			0	1	–	–	–
$90\%$ CR	% within	$5~\deg ^2$	$<1$	1–5	1–4	3–7	23–30
		$20~\deg ^2$	$<1$	7–14	12–21	14–22	65–73
	Median/$\deg ^2$		460–530	230–320	120–180	110–180	9–12
Searched area	% within	$5~\deg ^2$	4–6	15–21	20–26	23–29	62–67
		$20~\deg ^2$	14–17	33–41	42–50	44–52	87–90

Ranges reflect the uncertainty in the detector noise spectra shown in Fig. 1. The burst ranges assume standard-candle emission of $10^{-2}\,M_\odot c^2$ in gravitational waves at $150~\mathrm {Hz}$ and scale as $E_\mathrm {GW}^{1/2}$, so it is greater for more energetic sources (such as binary black holes). The binary neutron star (BNS) localization is characterized by the size of the $90\%$ credible region (CR) and the searched area. These are calculated by running the BAYESTAR rapid sky-localization code (Singer and Price 2016) on a Monte Carlo sample of simulated signals, assuming senisivity curves in the middle of the plausible ranges (the geometric means of the upper and lower bounds). The variation in the localization reflects both the variation in duty cycle between 70% and $75\%$ as well as Monte Carlo statistical uncertainty. The estimated number of BNS detections uses the actual ranges for 2015–2016 and 2017–2018, and the expected range otherwise; future runs assume a 70–$75\%$ duty cycle for each instrument. The BNS detection numbers also account for the uncertainty in the BNS source rate density (Abbott et al. 2017i). Estimated BNS detection numbers and localization estimates are computed assuming a signal-to-noise ratio greater than $\sim 12$. Burst localizations are expected to be broadly similar to those derived from timing triangulation, but vary depending on the signal bandwidth; the median burst searched area (with a false alarm rate of $\sim 1~\mathrm {yr^{-1}}$) may be a factor of $\sim 2$–3 larger than the values quoted for BNS signals (Essick et al. 2015). No burst detection numbers are given, since the source rates are currently unknown. Localization numbers for 2016–2017 include Virgo, and do not take into account that Virgo only joined the observations for the latter part the run. The 2024+ scenario includes LIGO-India at design sensitivity

Equivalent (but not directly comparable) results for bursts are found in Essick et al. (2015). Specific results depend upon the waveform morphology used, but the median searched area is $\sim 1$–2 times larger than for BNS signals; part of this difference is due to the burst study using a less-stringent FAR threshold of $\sim 1~\mathrm {yr^{-1}}$. The distribution of searched areas for four waveform morphologies are shown in Fig. 7.

**Fig. 8:**
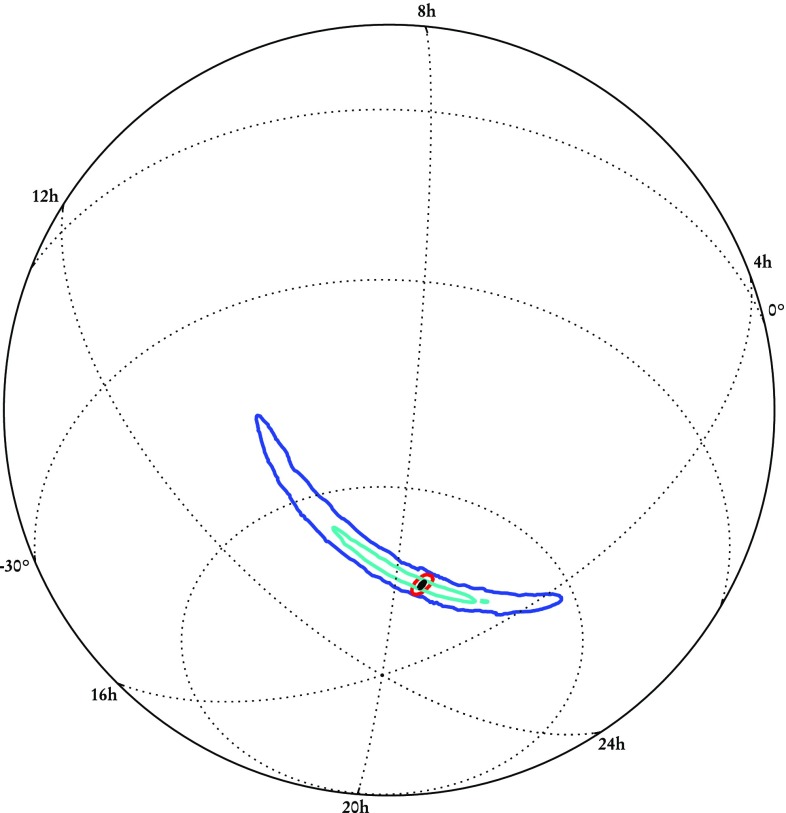
Sky localization of a signal with parameters consistent with those for GW150914. The lines enclose the $90\%$ credible regions with different detector networks. Dark blue is for the O1 two-detector network; light blue is for the same Hanford–Livingston network at design sensitivity; red is for the three-detector network including Virgo, with all detectors at early sensitivity, similar to what was expected for O2, and black is for the three detector network at design sensitivity. The plot is an orthographic projection with right ascension measured in hours and declination measured in degrees. Results taken from Gaebel and Veitch (2017)

The localizations of GW150914, GW151226 and LVT151012 exhibit the characteristic broken arc for a two-detector network (Abbott et al. 2016i, c). The $90\%$ credible regions are $230~\mathrm {deg^2}$, $850~\mathrm {deg^2}$ and $1600~\mathrm {deg^2}$ respectively (Abbott et al. 2016c). The sky localization for a CBC signal consistent with the properties of GW150914 is shown in Fig. 8. This shows the localization with the two-detector O1 network as well as with other detector network configurations (Gaebel and Veitch 2017).

The poor localization from a two-detector network makes follow-up challenging. The electromagnetic follow-up effort for GW150914 is described in Abbott et al. (2016i), Abbott et al. (2016n), and the search for coincident neutrinos is described in Adrian-Martinez et al. (2016), Albert et al. (2017c).

### 2016–2017 run (O2): aLIGO joined by AdV

This was an approximately nine-month run with three detectors for the second part of the run. The aLIGO performance was expected to be similar to the mid band in Fig. 1, with a BNS range of 80–120 $\mathrm {Mpc}$, and a burst range of 60–$75~\mathrm {Mpc}$ for $E_\mathrm {GW}=10^{-2}\,M_\odot c^2$: the achieved BNS range is towards the lower part of this band, around 60–100 $\mathrm {Mpc}$ (Abbott et al. 2017f). The AdV range was anticipated to be within the early band in Fig. 1, approximately 20–65 $\mathrm {Mpc}$ for BNS and 20–$40~\mathrm {Mpc}$ for bursts. On 1 August 2017, AdV joined O2 with a BNS range of 25–30 $\mathrm {Mpc}$.

The potential improvement in sky localization from the addition of a third detector is illustrated in Fig. 8.

Anticipated BNS sky localization for 2016–2017 (in addition to 2015–2016) was investigated in Singer et al. (2014). This assumed a noise curve which lies in the middle of the mid range in Fig. 1 for aLIGO—the mid curve specified in Barsotti and Fritschel (2012)—and the geometric mean of the upper and lower bounds of the mid region in Fig. 1 for AdV. The distribution of results is shown in Fig. 6. In Table 3, we give results for an astrophysically-motivated BNS population, with an SNR threshold of 12, assuming a three-detector network with each detector having an individual duty cycle of 70–$75\%$. The results are calculated using bayestar. The median $90\%$ credible region is 230–$320~\mathrm {deg}^2$, and 7–$13\%$ of events are expected to have $\mathrm {CR}_{0.9}^\mathrm {BNS}$ smaller than $20~\mathrm {deg}^2$. The searched area is smaller than $20~\mathrm {deg}^2$ for 33–$41\%$ of events and smaller than $5~\mathrm {deg}^2$ for 16–$21\%$. The burst study (Essick et al. 2015) gives approximately equivalent results, producing median searched areas a factor of $\sim 2$–3 larger than the BNS results; these results are shown in Fig. 7.

GW170104 (Abbott et al. 2017f) and GW170608 (Abbott et al. 2017g) were detected prior to Virgo joining O2. Therefore, like the O1 events, they have large $90\%$ credible areas of $1200~\mathrm {deg^2}$ and $860~\mathrm {deg^2}$ respectively. The addition of Virgo made a significant impact for GW170814 (Abbott et al. 2017h) and GW170817 (Abbott et al. 2017i). For GW170814, the initial bayestar $90\%$ credible area is reduced from $1160~\mathrm {deg^2}$ to $100~\mathrm {deg^2}$ with the addition of Virgo, and the final LALInference three-detector localization is $60~\mathrm {deg^2}$. For GW170817, the initial bayestar $90\%$ credible area is reduced from $190~\mathrm {deg^2}$ to $31~\mathrm {deg^2}$ with the addition of Virgo, and the final LALInference three-detector localization is $28~\mathrm {deg^2}$. The inclusion of the third detector to the network enhances localization whether or not it detects the signal, provided that it could detect the signal, as the observed amplitude constrains the source position. As a result of being observed with a three-detector network, and its high SNR, GW170817 has the best GW localization to date.

GW170817 is the first GW signal to have a confirmed electromagnetic counterpart. The discoveries associated with this detection are highlighted in Sect. 3. An overview of the extensive multi-messenger observations accompanying GW170817 is given in Abbott et al. (2017k).

### 2018–2019 run (O3): aLIGO 120–170 Mpc, AdV 65–85 Mpc

This is envisioned to be a year long run with three detectors. The aLIGO and AdV sensitivities will be similar to the late and mid bands of Fig. 1 respectively, with BNS ranges of 120–170 $\mathrm {Mpc}$ and 65–85 $\mathrm {Mpc}$, and burst ranges of 75–90 $\mathrm {Mpc}$ and 40–50 $\mathrm {Mpc}$ for $E_\mathrm {GW}=10^{-2}\,M_\odot c^2$. This gives an expected update 1–50 BNS detections. Both the range and the typical sky localization should increase relative to the 2016–2017 run. Table 3 gives bayestar localizations assuming detector sensitivities which are the geometric means of the upper and lower bounds of the relevant bands in Fig. 1. The median $90\%$ credible region is 120–$180~\mathrm {deg}^2$, and 12–$21\%$ of events are expected to have $\mathrm {CR}_{0.9}^\mathrm {BNS}$ smaller than $20~\mathrm {deg}^2$.

### 2020+ runs: aLIGO 190 Mpc, AdV 65–125 Mpc

At this point we anticipate extended runs with the detectors at or near design sensitivity. The aLIGO detectors are expected to have a sensitivity curve similar to the design curve of Fig. 1. AdV may be operating similarly to the late band, eventually reaching the design sensitivity circa 2021. Potential localization for a GW150914-like BBH signal is shown in Fig. 8. The fraction of signals localized to areas of a few square degrees is increased compared to previous runs. This is due to the much larger detector bandwidths, particularly for AdV, as well as the increased sensitivity of the network; see Fig. 1.

### 2024+ runs: aLIGO (including LIGO-India) 190 Mpc, AdV 125 Mpc, KAGRA 140 Mpc

The five-site network incorporating LIGO-India at design sensitivity would have both improved sensitivity and better localization capabilities. The per-year BNS search volume increases giving an expected 11–180 BNS detections annually. The addition of more detector sites leads to good source localization over the whole sky (Schutz 2011; Veitch et al. 2012; Nissanke et al. 2013; Rodriguez et al. 2014). Table 3 gives bayestar localizations for an astrophysical population of BNSs, assuming design sensitivity and a 70–$75\%$ duty cycle for each detector. The median $90\%$ credible region is 9–$12~\mathrm {deg}^2$, 65–$73\%$ of events are expected to have $\mathrm {CR}_{0.9}^\mathrm {BNS}$ smaller than $20~\mathrm {deg}^2$, and the searched area is less than $20~\mathrm {deg}^2$ for 87–$90\%$.

## Conclusions

We have presented possible observing scenarios for the Advanced LIGO, Advanced Virgo and KAGRA network of GW detectors, with emphasis on the expected sensitivities and sky-localization accuracies. This network began operation in September 2015 with the two LIGO detectors. Virgo joined the network in August 2017, dramatically improving sky localization. With a four- or five-site detector network at design sensitivity, we may expect a significant fraction of GW signals to be localized to within a few square degrees by GW observations alone.

The first BBH detection was made promptly after the start of observations in September 2015; they are the most commonly detected GW source, but are not a promising target for multi-messenger observations. GW detections will become more common as the sensitivity of the network improves. The first BNS coalescence was detected in August 2017. This was accompanied by observations across the electromagnetic spectrum (Abbott et al. 2017k). Multi-messenger observations of BNSs provide new insights into binary evolution, nuclear physics, cosmology and gravitational physics.

Optimizing the multi-messenger follow-up and source identification is an outstanding research topic (e.g., Abadie et al. 2012b; Aasi et al. 2014a; Kasliwal and Nissanke 2014; Singer et al. 2014; Cannon et al. 2012; Evans et al. 2016a; Gehrels et al. 2016; Ghosh et al. 2016; Chan et al. 2017; Rana et al. 2017; Salafia et al. 2017; Patricelli et al. 2018). Triggering of focused searches in GW data by electromagnetically-detected events can also help in recovering otherwise hidden GW signals (Aasi et al. 2014c). Multi-messenger follow-up of GW candidates *may* help confirm GW candidates that would not be confidently identified from GW observations alone. However, such follow-ups need to deal with large position uncertainties, with areas of many tens to thousands of square degrees. This is likely to remain the situation until late in the decade.

The purpose of this article is to provide information to the astronomy community to facilitate planning for multi-messenger astronomy with advanced GW detectors. *While the scenarios described here are our best current projections, they will evolve as detector installation and commissioning progress.* We will therefore update this article regularly.

## A Changes between versions

Since publication of the previous version (Aasi et al. 2016), several updates to the document have been made. The most significant changes are the inclusion of details regarding KAGRA, and results from O1 and O2, including GW170817, the first detection with a unambiguous multi-messenger counterpart (Abbott et al. 2017k). The key differences are outlined below.

### A.1 Updates to detector commissioning

The plausible detector scenarios remain largely unchanged, but details of O1 and O2 have been updated. Specific updates to the detector scenarios are:

1. The addition of KAGRA to Figs. 1 and  2.
2. Table 1 has been added which includes BNS ranges and $30\,M_{\odot }$+$30\,M_{\odot }$ BBH ranges.
3. The O1 sensitivity has been updated following the completion of the run (12 September 2015–19 January 2016). We believed that the O1 BNS range would plausibly be 40–80 $\mathrm {Mpc}$, and the actual range was 60–80 $\mathrm {Mpc}$.
4. The formal transition to O2 was 30 November 2016, and the run ended 25 August 2017. The run is approximately nine calendar months in duration, but includes a two-week break at the end of December and a three-week commissioning break in May. The achieved aLIGO BNS range was approximately 60–100 $\mathrm {Mpc}$ (Abbott et al. 2017f).
5. The AdV detector officially joined the O2 run on 1 August 2017. The achieved AdV BNS range was approximately 25–30 $\mathrm {Mpc}$.
6. As a consequence of the extended O2 run, the start of O3 is now expected to be in 2018, approximately a year after the end of O2. O3 is now planned to be a year in duration.
7. Figure 2 has been updated to show the current planned timeline.
8. Based on our experience in O1 and O2, we have revised our predicted duty cycles, we believe that single-detector duty cycles of 70–$75\%$ for extended runs are more realistic than the previous value of $80\%$.
9. We have updated the final observing scenario, the 2024+ case, to be a five-detector network including KAGRA.
10. Progress has been made towards establishing LIGO-India, with the the Indian government granting in-principle approval; however, it is still too early to give a definite timeline for the observing schedule. 2024 is the earliest we imagine it could be operational.
11. Some of the BNS ranges associated with different observing scenarios have changed as a result of using an updated calculation, including cosmological corrections. For all cosmological calculations, we assume a flat cosmology with Hubble parameter $H_0 = 67.9~\mathrm {km\,s^{-1}\,Mpc^{-1}}$, and density parameters $\Omega _\mathrm {m} = 0.3065$ and $\Omega _\Lambda = 0.6935$ (Ade et al. 2016). The sensitivity curves themselves have not been modified. Ranges are rounded to the nearest megaparsec below $15~\mathrm {Mpc}$, rounded to the nearest $5~\mathrm {Mpc}$ between $15~\mathrm {Mpc}$ and $1000~\mathrm {Mpc}$, and to the nearest $10~\mathrm {Mpc}$ above $1000~\mathrm {Mpc}$.

### A.2 Updates to data analysis

In addition to the progress made with regards to the detectors, there have also been significant advances from analysing the data. We now include results from O1 (Abbott et al. 2016c, q, 2017b) and initial results from O2; these include the first detections of BBH and BNS systems (Abbott et al. 2016k, g, c, 2017f, g, h, i). Multi-messenger searches for counterparts to these detections are described in Abbott et al. (2016i, 2016n); Adrian-Martinez et al. (2016); Albert et al. (2017c); Abbott et al. (2017k). Specific updates that have been made are:

1. Figure 3 has been updated to include O1 results; the surrounding text has been updated to discuss the detection pipelines used in O1 (Abbott et al. 2016e, c, l, 2017b).
2. Results using the BayesWave algorithm (Bécsy et al. 2016), appropriate for O1, have been included in Fig. 7.
3. Figure 8 shows a sky localization, created using current parameter-estimation techniques, illustrating how localization improves for different network configurations for a GW150914-like signal (Gaebel and Veitch 2017).
4. Table 3 now includes numbers summarising results from O1 and the new 2024+ scenario including KAGRA. Sky localization numbers for are now calculated using bayestar (Singer and Price 2016) for all epochs.
5. BNS merger rates have been updated following the observation of GW170817. The post-detection range is 320–$4740~\mathrm {Gpc^{-3}\,yr^{-1}}$ (Abbott et al. 2017i), consistent with the previous expectation of 10–$10^{4}~\mathrm {Gpc^{-3}\,yr^{-1}}$ (Abadie et al. 2010b).

We defer describing the full results of O2 until a future update.

## Footnotes

1 LIGO is short for Laser Interferometer Gravitational-Wave Observatory. KAGRA is named after the Japanese word for traditional sacred music and dance for the gods *kagura*; the name has a secondary meaning as an abbreviation for KAmioka GRavitational-wave Antenna. Virgo is not an acronym, and is not written in all caps.

2 The detection of GW150914 occurred in the engineering run ER8 immediately preceding the formal start of O1.

3 Details of the GCN are available from gcn.gsfc.nasa.gov, and archives of messages for GW150914 (gcn.gsfc.nasa.gov/other/G184098.gcn3), LVT151012 (gcn.gsfc.nasa.gov/other/G197392.gcn3), GW151226 (gcn.gsfc.nasa.gov/other/G211117.gcn3), GW170104 (gcn.gsfc.nasa.gov/other/G268556.gcn3), GW170608 (gcn.gsfc.nasa.gov/other/G288732.gcn3), GW170814 (gcn.gsfc.nasa.gov/other/G297595.gcn3) and GW170817 (gcn.gsfc.nasa.gov/other/G298048.gcn3) are now publicly available.

4 More information about the follow-up program is available at gw-astronomy.org/wiki/LV_EM/TechInfo.

5 The *p* value is distinct from the probability that a trigger is not a real astrophysical GW signal. The *p* value assumes that the data contain no signals, whereas the probability of there being a GW must include the hypothesis that there is an astrophysical signal. To calculate the probability that a trigger is a real signal (or not) requires an extra layer of inference, folding in both our knowledge of the distribution of triggers, assumptions about the signal distribution (such as that sources are uniformly distributed in volume), and knowledge and assumptions about the merger rate per unit volume for a class of sources. A method for doing this is described in Abbott et al. (2016p, 2016o, 2016c).

6 Strain data from published probable events in O1 and O2 are publicly available from the LIGO Open Science Center losc.ligo.org (Vallisneri et al. 2015). Full data from O1 are also available, and data from other observing runs will be added in the future.

7 A data release of example three-dimension localizations in this format, constructed using results from bayestar and LALInference for BNS signals, is available from dcc.ligo.org/P1500071/public/html.

8 This assumes a black hole mass of $5\,M_\odot$.

9 This study used noise from the sixth science run of initial LIGO, recolored to the expected O1 sensitivity curve. The source catalog, as well as the analysis pipeline, is shared with Singer et al. (2014).
